# SOX30 Governs Synaptonemal Complex Assembly and Homologous Recombination in Male Meiosis

**DOI:** 10.1111/cpr.70158

**Published:** 2025-12-30

**Authors:** Kangle Liu, Wenfeng Zhang, Xiao Jiang, Jianping Chen, Lei Zhu, Zhonghao Zhang, Jing Gu, Lulu Guo, Lin Ao, Qing Chen, Lei Sun, Yuhan Hu, Xin Wang, Yaxin Liu, Jia Cao, Fei Han, Jinyi Liu

**Affiliations:** ^1^ Institute of Toxicology, College of Preventive Medicine, Army Medical University Chongqing China; ^2^ State Key Lab of Trauma and Chemical Poisoning, and Key Lab of Medical Protection for Electromagnetic Radiation, Ministry of Education of China, Army Medical University Chongqing China; ^3^ Chongqing Municipal Key Laboratory of Hygiene Toxicology of Higher Education Chongqing China; ^4^ Joint International Research Laboratory of Reproduction and Development of the Ministry of Education, School of Public Health, Chongqing Medical University Chongqing China

**Keywords:** homologous recombination, meiosis, SOX30, spermatogenesis, synaptonemal complex

## Abstract

Meiosis, a specialised form of cell division, is essential for sexual reproduction, which requires the proper formation of synaptonemal complex (SC) and homologous recombination (HR). However, the regulatory mechanisms underlying these processes remain incompletely understood. Here, we demonstrate that SOX30 is a key transcriptional regulator of male meiotic synapsis and recombination. In *Sox30*‐knockout mice, zygotene spermatocytes accumulate with synapsis defects. SOX30 deficiency disrupts the SC central element components SYCE1, SYCE2, and TEX12 distribution. Furthermore, disrupted γ‐H2AX distribution reveals impaired DNA double‐strand break repair and the persistence of recombination proteins RAD51 and RPA2 in late spermatocytes confirms defective homologous recombination repair (HRR) which results in reduced crossover formation in *Sox30*‐knockout mice spermatocytes. Mechanistically, SOX30 directly binds to SYCE1/SYCE2 promoters to modulate their transcription, thereby regulating SC assembly and HRR. Restoring SOX30 expression effectively rescues meiotic defects. Importantly, transcriptome co‐expression analysis in non‐obstructive azoospermia (NOA) testes identifies SOX30 as a central regulator of NOA transcriptional networks. Collectively, these findings underscore SOX30's crucial role in meiotic synapsis and recombination, highlighting its therapeutic potential for NOA.

## Graphical abstract

The transcription factor SOX30 directly binds promoter regions of SYCE1 and SYCE2 to mediate their transcriptional activation, thereby enabling proper assembly of central elements within the synaptonemal complex. Structural destabilisation of the synaptonemal complex in Sox30 KK spermatocytes triggers synaptic discontinuity, impairs homologous recombination repair, and reduces MLH1‐marked crossover formation leading to meiotic progression arrest at the zygotene.

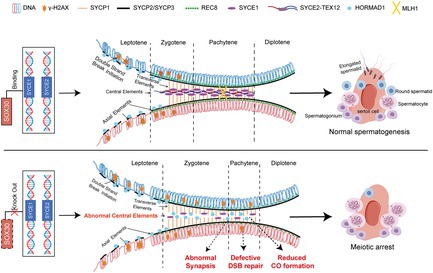

## 1 Introduction

Meiosis is a specialised cell division process involving two consecutive divisions (meiosis I and II), which together produce haploid gametes following a single round of DNA replication [1]. The initial meiotic division (meiosis I) includes four key stages: Prophase I, Metaphase I, Anaphase I, and Telophase I. Prophase I is the longest and most complex phase, which can be subdivided into five sequential substages: leptotene, zygotene, pachytene, diplotene, and diakinesis [2]. During Prophase I, homologous chromosomes start pairing and triggering large‐scale programmed DNA double‐strand breaks (DSBs). Critically, DSB repair of homologous chromosomes occurs simultaneously with synapsis and shows interdependence [3]. Synapsis initiates when 3′‐end overhangs from DSBs invade homologous DNA sequences to establish recombination intermediates. This process engages the axial elements of the synaptonemal complex (SYCP3, SYCP2), which are bridged by SYCP1 alongside central element components (SYCE1, SYCE2, SYCE3, TEX12) [4, 5, 6]. Complete synapsis is achieved when the DSB repair intermediates are resolved into crossover (CO), and intact SC will stabilise highly condensed chromosomes [7]. The precise synchronisation of DSB repair and homologous chromosome synapsis proves essential for ensuring proper segregation during meiosis I. Establishing the correct SC structure is fundamental to this coordinated process. During leptotene, homologous chromosomes commence pairing, thereby inducing large‐scale programmed DSBs. Alongside DSB sites, DNA ends are enzymatically processed into 3′ single‐stranded overhangs that invade double‐stranded DNA of homologous chromosomes, giving rise to recombination intermediates. During progression of DSB repair, central element proteins organise between the axial elements of paired homologous chromosomes to construct bridging structures spanning the two axes. This assembly process promotes the synapsis from zygotene to pachytene and finally forms a complete SC and a close homologous chromosome synapsis. The DSB repair intermediate is decomposed into crossover, which triggers SC disintegration and marks the transition from pachytene to diplotene and promotes extensive chromosome condensation. In general, synapsis and DSB repair are interdependent core events in meiosis prophase I, which is essential for accurate homologous chromosome separation and genetic recombination; its destruction can lead to defects in germ cell development or genetic abnormalities.

To date, extensive studies have identified numerous genes associated with meiotic DSB repair and synapsis. Deletion of any such gene typically induces meiotic and spermiogenic abnormalities [3, 4, 5]. Furthermore, these genes are regulated by complex transcriptional networks, and male and female meiosis have different regulatory mechanisms [8, 9]. Nevertheless, current knowledge regarding transcription factors constituting this network remains incomplete, particularly in male meiosis. Although transcription factors such as MEIOSIN and MYBL1 have been characterised as stage‐specific meiotic regulators, current knowledge regarding the transcriptional network components remains fragmentary [10, 11]. Notably, numerous critical molecules within the male meiotic regulatory network await further characterization.

*SOX30*, a member of the SRY (sex‐determining region Y)‐box (SOX) family, is a transcription factor characterised by a highly conserved HMG (high mobility group)‐box domain responsible for DNA‐binding [12]. In recent years, significant advances have been made in understanding its function, with growing attention focused on its critical role in spermatogenesis [13, 14, 15, 16, 17, 18, 19, 20]. Notably, SOX30 exhibits specific and high expression in the testis, and its expression levels progressively increase postnatally. In contrast, it is minimally expressed in the ovary and other tissues, which underscores its close association with male sexual development and the maintenance of testicular function [15, 16]. Our previous studies have shown that the promoter region of *SOX30* is significantly hypermethylated and low expressed in the testicular tissue of NOA patients, and the degree of methylation correlates positively with disease severity [21, 22]. A gene‐knockout mouse model revealed that the absence of *Sox30* results in male mouse infertility, with spermiogenesis arrested at the round sperm stage, and meiotic arrest occurring as the mice age [23]. Single‐cell sequencing of the testicular tissue from *Sox30‐*knockout (KO) mice indicated that *Sox30* deficiency causes spermatocyte arrest at the early phase of meiosis I [24]. Collectively, these findings identify SOX30 as a key transcription factor of the transcriptional regulatory network governing male meiosis. Nevertheless, the precise role and mechanisms of SOX30 in meiosis remain unclear.

This study shows SOX30 loss disrupts meiotic synapsis and homologous recombination, which causes zygotene spermatocyte accumulation. Although SYCP3/SYCP1 localization is unaffected, central SC components (SYCE1, SYCE2, TEX12) exhibit abnormal localization and reduced expression. Persistent DSBs and elevated RPA2/RAD51 levels in late‐stage spermatocytes indicate impaired DSB repair, which results in reduced MLH1 foci. Mechanistically, SOX30 directly binds *Syce1/Syce2* promoters to modulate their transcription, thereby controlling SC assembly and HRR. Restoration of SOX30 significantly rescues synapsis and DSB defects in knockout mice, while transcriptomic analysis of NOA patients identifies SOX30 as a core regulator of spermatogenesis. In summary, SOX30 governs SC integrity and HRR via transcriptional regulation; its dysfunction leads to meiotic failure.

## 2 Results

### 2.1 SOX30 Deficiency Causes Meiotic Arrest in Spermatocytes

To elucidate the expression dynamics of SOX30 during spermatogenesis, we performed immunofluorescence co‐staining of SOX30 and the spermatocyte marker SYCP3 in testicular tissues (Figure S1A) and analysed publicly available single‐cell RNA sequencing datasets (http://malehealthatlas.cn/) from human and mouse testes [25] (Figure S1B,C). In both species, SOX30 expression initiates at the zygonema spermatocyte stage, gradually increases thereafter, and remains elevated throughout spermiogenesis. This spatiotemporal expression pattern was further confirmed by immunofluorescence (Figure S1A). Moreover, transcriptomic analysis of testes from *Sox30* knockout (KO) mice revealed significant differential expression of key genes involved in meiosis (Figure S1D). Gene Set Enrichment Analysis (GSEA) further identified strong enrichment for terms such as ‘negative regulation of meiotic nuclear division’ (Figure S1E) and ‘meiotic cell cycle’ (Figure S1F). These results suggest the potential regulatory role of SOX30 in the meiotic process of male mice.

**FIGURE 1:**
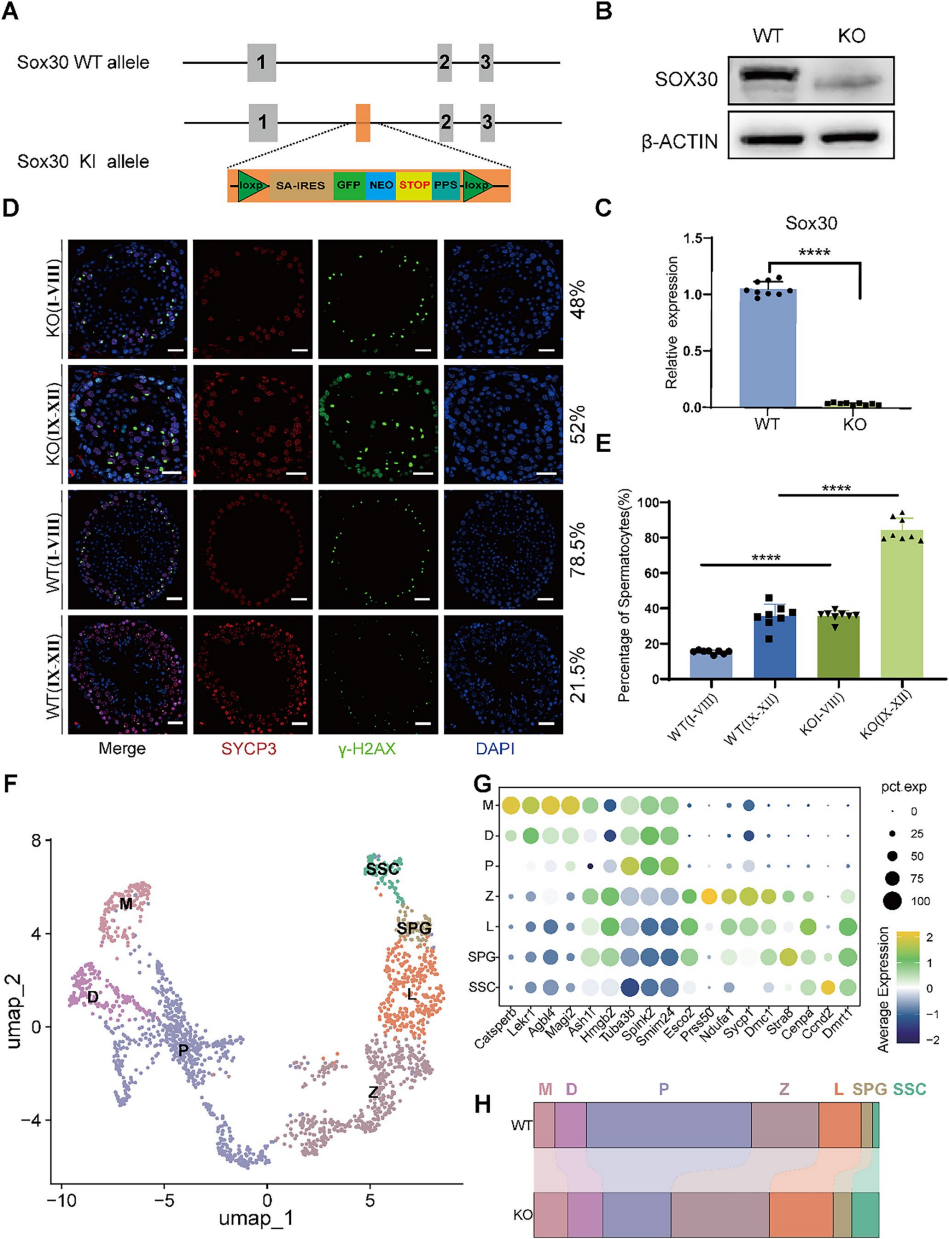
*SOX30* knockout disrupts meiotic progression and spermatogenic homeostasis. (A) Schematic of the *Sox30* knockout strategy using insertion of a LoxP‐splice acceptor (SA)‐IRES‐GFP‐Neo‐STOP‐polyA‐LoxP cassette in exon 12. (B, C) Validation of SOX30 ablation by Western blot (B) and quantitative RT‐PCR (C) showing complete loss of SOX30 protein and mRNA in testes (WT: Wild‐type; KO: Knockout; *n* = 9, ****p < 0.0001, Student's *t*‐test). (D) Immunofluorescence of testicular sections stained for SYCP3 (red) and γ‐H2AX (green) in WT and *Sox30* KO mice. Scale bar: 20 μm. (E) Quantification of spermatocyte proportions in seminiferous tubules (*n* = 8 tubules/group; ****p < 0.0001, Student's *t* test). (F) UMAP projection of integrated single‐cell RNA‐seq data of spermatogonia and spermatocytes from both WT and KO testes, illustrating the overall landscape and annotation of major spermatogenic cell types. The separate UMAP projections by genotype are provided in Figure S2C for direct comparison. (D: diplotene; L: leptotene; M: metaphase; P: pachytene; SPG: spermatogonial; SSC: spermatogonial stem cell; Z: zygotene) (G) dot plot showing expression of stage‐specific markers across cell clusters (dot size: percentage; colour: average expression). (H) Bar graphs comparing meiotic stage proportions between WT and KO groups. *Sox30* deficiency causes accumulation of zygotene spermatocytes and depletion of pachytene/diplotene cells.

To explore the specific role and mechanism of SOX30 within the transcriptional regulation network of male meiosis, we generated a *Sox30* KO mouse model (Figure 1A). The successful establishment of the knockout model was confirmed through Western blot (WB) and quantitative PCR (qPCR) analyses, which demonstrated a complete absence of SOX30 (Figure 1B,C). The band observed in the KO lane in Figure 1B is non‐specific, as determined by its distinct electrophoretic mobility compared to the authentic SOX30 band in WT samples. This non‐specific signal likely results from antibody cross‐reactivity. Through immunofluorescence analysis of testicular tissues in wild‐type (WT) and *Sox30* knockout mice, significant alterations in spermatogenesis were observed. Following *Sox30* knockout, the distribution of seminiferous tubule stages shifted markedly. Tubules corresponding to wild‐type stages I–VIII decreased from 78.5% to 48% of the total, while those resembling stages IX–XII increased from 21.5% to 52% (Figure 1D). Concurrently, spermatocyte proportions surged dramatically: in tubules analogous to wild‐type stages I–VIII, spermatocytes rose from 22%–46% to 78%–94%, and in tubules resembling stages IX–XII, they increased from 13%–17% to 29%–39% (Figure 1E). These observations reveal that SOX30 plays a critical role in regulating both the stage distribution of seminiferous tubules and the number of spermatocytes during spermatogenesis.

We systematically extracted and analysed spermatogonia and spermatocyte subsets in our single cell data to determine the stage of meiotic arrest caused by *Sox30* knockout. Following dimensionality reduction clustering, we integrated dynamic expression patterns of marker genes from publicly available datasets to classify spermatogonia and spermatocytes at various stages with higher resolution (Figure 1F,G, and Figure S2A). To present this annotated cellular landscape, we show the integrated UMAP projection of both genotypes in Figure 1F. For direct visual comparison of cell population distributions, the separate UMAP plots for WT and KO are provided in Figure S2C. Furthermore, developmental trajectory analysis of these defined cell types confirmed their distribution along the spermatogenesis trajectory (Figure S2B). Upon defining the cell types, we found that *Sox30* knockout led to an accumulation of spermatogonia and zygotene spermatocytes, alongside a reduction in the proportion of pachytene spermatocytes (Figure 1H, Figure S2C).

### 2.2 SOX30 Deficiency Causes Defective Homologous Chromosome Synapsis

**FIGURE 2:**
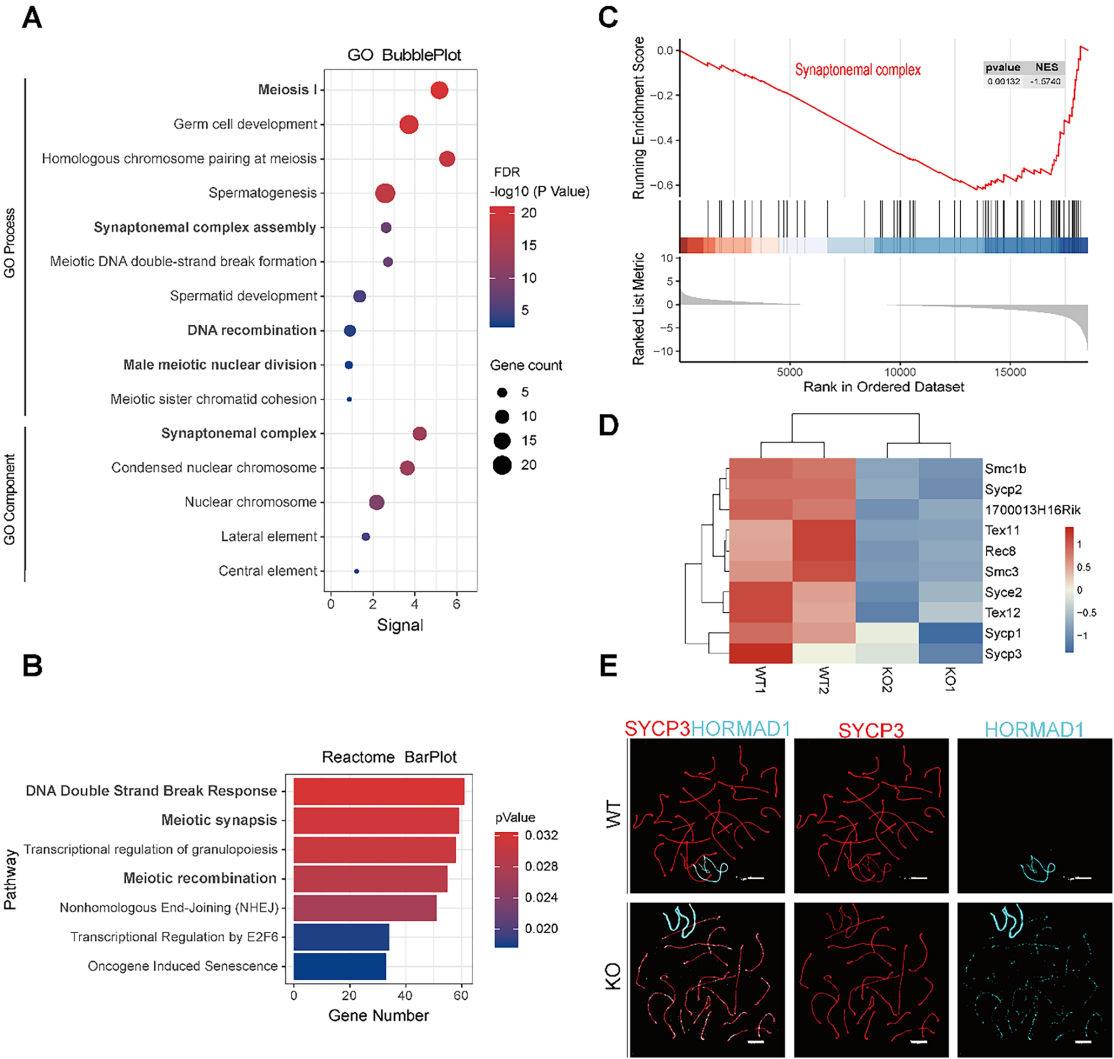
Sox30 deficiency causes defective homologous chromosome synapsis. (A) GO enrichment analysis of SOX30 related genes in STRING database. (B) REACTOME pathway analysis of SOX30‐interacting genes. (C) GSEA plot showing significant downregulation of ‘synaptonemal complex’ genes (NES = −1.574, FDR < 0.01) in Sox30 KO testis. (D) Heatmap of differentially expressed SC‐associated genes in Sox30 KO vs. WT testes. (E) Chromosome spread immunofluorescence co‐staining for HORMAD1 (cyan, unsynapsed axes) and SYCP3 (red, lateral elements) in WT and Sox30 KO pachytene spermatocytes. KO cells exhibit discontinuous synapsis and HORMAD1 retention. (scale bar: 3 μm).

To investigate the mechanism underlying SOX30‐induced accumulation of zygotene spermatocytes, we performed enrichment analysis of genes interacting with or co‐expressed with SOX30 using the STRING database. The analysis revealed significant enrichment of meiosis‐related biological processes, including ‘meiosis I’ and ‘meiotic sister chromatid segregation’, as well as synapsis‐associated terms such as ‘synaptonemal complex’ (Figure 2A). Enrichment analysis of predicted SOX30 interacting proteins from the Reactome database revealed significant associations with meiotic progression and homologous recombination (HR)‐related processes, including ‘DNA Double‐Strand Break Response’, ‘Meiotic Synapsis’, and ‘Meiotic Recombination’ (Figure 2B). Gene Set Enrichment Analysis (GSEA) of transcriptomic data from *Sox30* KO mice further revealed significant enrichment of ‘Synaptonemal complex’‐related terms (Figure 2C). Notably, key SC‐associated genes such as Syce2, Sycp2 and Smc1b were significantly downregulated in the transcriptome data (Figure 2D), indicating that SOX30 may regulate the formation of the meiotic SC.

HORMAD1 localises to unsynapsed or desynapsed chromosome axes [26]. To assess the effect of *SOX30* deletion on synapsis, chromosome spreading assays were prepared and co‐stained for SYCP3 and HORMAD1. In *Sox30* KO mice, we observed scattered dot‐like signals along the autosome axes of pachytene spermatocytes (the stage when synapsis completes), whereas no such signals were detected in WT mice (Figure 2E). These HORMAD1‐positive foci indicate synaptic defects, confirming that *Sox30* deficiency disrupts the synapsis process evidenced by persistent unsynapsed regions on the autosome axes.

### 2.3 SOX30 Deficiency Leads to Abnormal Localization of the Central Component of Spermatocyte SC

**FIGURE 3:**
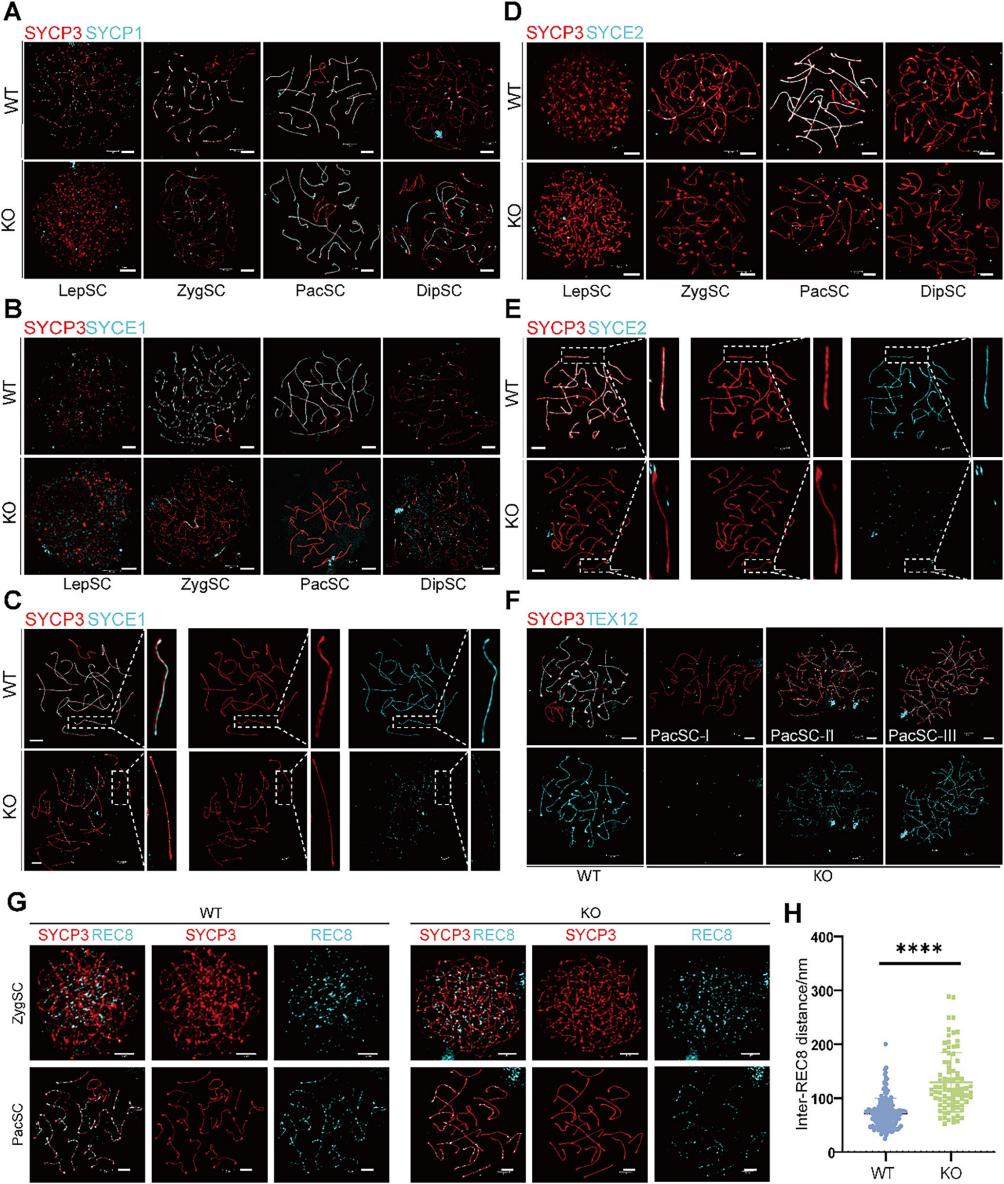
The structure of SC of spermatocytes in WT and *Sox30* KO mice. (A) Co‐immunofluorescence stain of SYCP3 (red) and SYCP1 (cyan) in chromosome spreads of spermatocytes from WT and KO mice (scale bar: 3 μm). (B, C) Co‐immunofluorescence stain of SYCP3 (red) and SYCE1 (cyan) in chromosome spreads of spermatocytes from WT and KO mice and the magnified view (scale bar: 3 μm). (D, E) Co‐immunofluorescence stain of SYCP3 (red) and SYCE2 (cyan) in chromosome spreads of spermatocytes from WT and KO mice and the magnified view (scale bar: 3 μm). (F) Co‐immunofluorescence stain of SYCP3 (red) and TEX12 (cyan) in chromosome spreads of spermatocytes from WT and KO mice (scale bar: 3 μm). (G) REC8 (cyan) and SYCP3 (red) co‐localization patterns in Sox30 KO spermatocytes. KO cells show discontinuous REC8 linear arrays (arrowheads) compared to WT. Scale bar: 3 μm. (H) Quantification of inter‐REC8 focus distances. Sox30 KO cells exhibit increased spacing (****p < 0.0001, *n* = 167 cells from 3 mice for WT group, *n* = 81 cells from 3 mice for KO group, Student *t* test). DipSC: diplotene; LepSC: leptotene; PacSC: pachytene; ZygSC: zygotene.

To further explore the specific effect of *Sox30* deletion on the structure of the SC, we examined each component of the SC using chromosome spreading assay combined with immunofluorescence co‐staining and super‐resolution microscopy. We analysed SYCP3, a component of the SC lateral elements, and SYCP1, a transverse filament protein of the SC. The results suggest that *Sox30* deletion does not impair the proper assembly of SYCP3 and SYCP1 (Figure 3A). We extended our analysis to other components of the SC. Immunofluorescence co‐staining was performed for the central components SYCE1, SYCE2, TEX12, along with SYCP3, followed by structural analysis using super‐resolution microscopy (Figure 3B,D,F). In *Sox30* KO spermatocytes, SYCE1 failed to form a continuous linear distribution along the chromosome axis as observed in WT mice, and instead, it exhibited a scattered dot‐like pattern, indicative of abnormal SC assembly (Figure 3C). Similarly, co‐staining of SYCE2 and SYCP3 revealed that SYCE2 was unable to localise properly to the chromosome axis in most spermatocytes from *Sox30* KO mice (Figure 3E). Nonetheless, a small subset of spermatocytes retained normal SYCE2 localization (Figure S3A). As part of the SYCE2–TEX12 complex, we further examined TEX12 localization. Our results identified three distinct localization patterns of TEX12 in testes following *Sox30* knockout (Figure 3F): type I showed complete absence of TEX12 signals, similar to the phenotype observed for SYCE2; type II showed discrete punctate staining along the chromosome axis, resembling the SYCE1 localization pattern; Type III showed a distribution pattern comparable to that seen in wild‐type mice. These findings suggest that SOX30 plays a critical role in regulating the proper localization and assembly of central SC components, particularly the SYCE1 and SYCE2–TEX12 complexes.

Although the central elements of the SC are not properly assembled in *Sox30* KO mice, SYCP1 staining still reveals a structural organisation comparable to that seen in wild‐type spermatocytes. Such a phenomenon is not uncommon. For example, it has been reported that HSF5 regulates the expression of SC components SYCP2 and SYCE3. However, in *Hsf5*‐deficient mice, SYCP1 localization remains unaffected [27]. This may be due to the ability of SYCP1 to retain self‐assemble and form synaptonemal structures even after gene knockout [28]. In addition, our previous study revealed that SOX30 transcriptionally regulates REC8, a critical component of the meiotic cohesin complex (Fig. S3B), which plays a vital role in the normal assembly of the meiotic SC [29]. Immunofluorescence co‐staining with SYCP3 demonstrated that the chromosomal distribution of REC8 was markedly sparser *in Sox30* KO mice compared to controls (Figure 3G). Moreover, the inter‐signal distances between adjacent REC8 foci were significantly increased in the knockout group compared to wild‐type (Figure 3H). WB analysis also confirmed a substantial reduction in REC8 at the protein level (Figure S3C). Collectively, these findings indicate that SOX30 deficiency leads to meiotic abnormalities in spermatocytes, primarily manifested as defective assembly of the SC's central components and a marked reduction in the integrity of the cohesin complex.

### 2.4 SOX30 Deficiency Leads to Impaired DSB Repair and Reduced CO Formation in Spermatocytes

**FIGURE 4:**
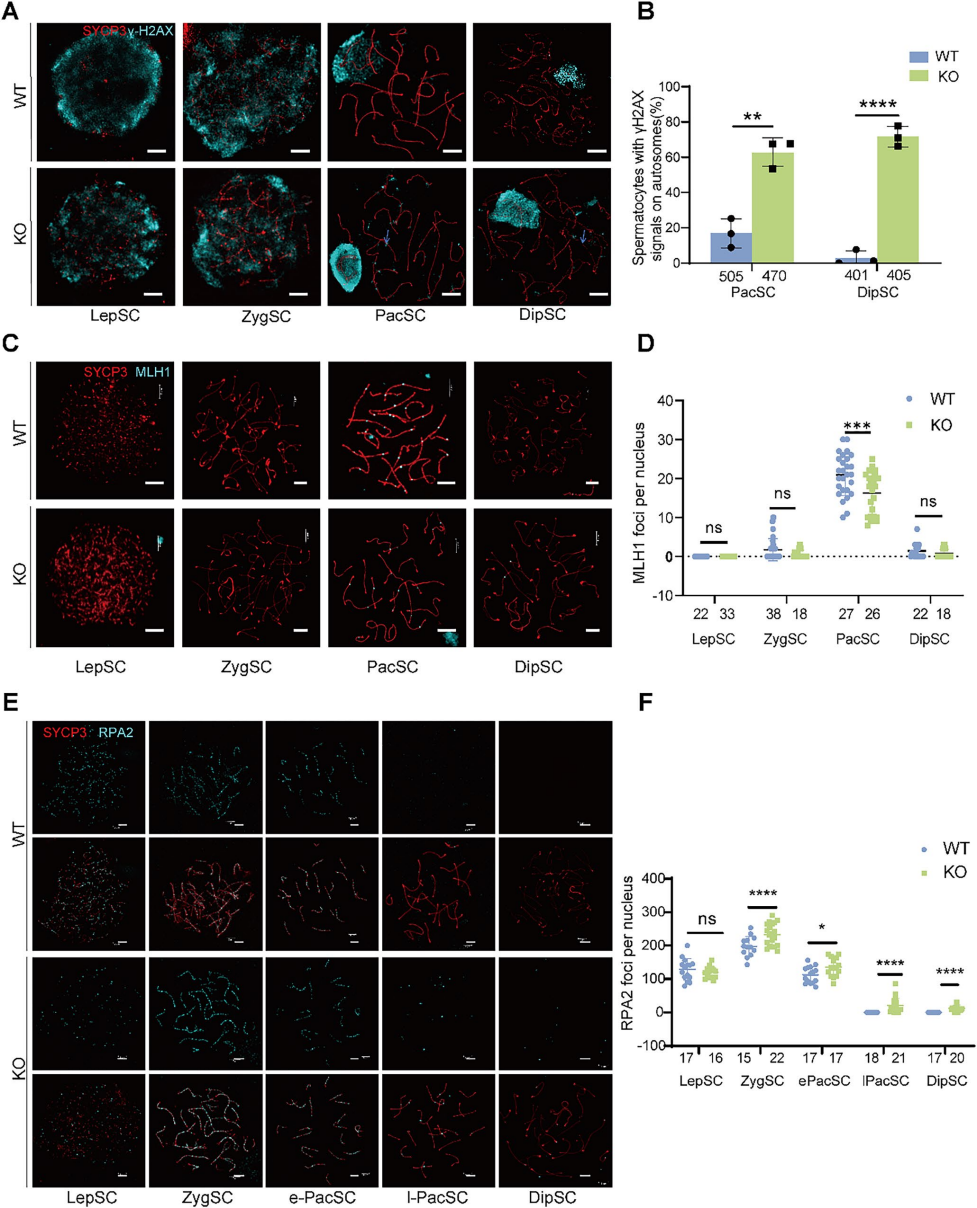
SOX30 deficiency disrupts meiotic DSB repair and crossover formation. (A) Chromosome spread immunofluorescence co‐staining for SYCP3 (lateral elements, red) and γH2AX (DNA damage foci, cyan) in WT and *Sox30* KO spermatocytes. Persistent γH2AX signals (arrowheads) on autosomes indicate unresolved double‐strand breaks (DSBs). Scale bar: 3 μm. (B) Quantification of pachytene/diplotene spermatocytes harbouring γH2AX^+^ autosomes. Numbers below the x‐axis labels indicate cells from 3 mice for analysed per group (**p < 0.01, ****p < 0.0001, Student's *t* test). (C) SYCP3 (red) and MLH1 (crossover sites, cyan) co‐staining in WT and *Sox30* KO cells. KO spermatocytes exhibit markedly reduced MLH1 foci. Scale bar: 3 μm. (D) Scatterplot comparing MLH1 foci counts across meiotic stages. Numbers below the *x*‐axis labels indicate cells from 3 mice for analysed per stage (ns: not significant, ***p < 0.001, Student's *t* test). (E) SYCP3 (lateral elements, red) and RPA2 (single‐strand DNA binding, cyan) co‐staining in Sox30 KO spermatocytes. Scale bar: 3 μm. (F) Scatterplot quantifying RPA2 foci counts across meiotic stages. Numbers below the *x*‐axis labels indicate cells from 3 mice for analysed per group. Only the foci co‐located with SYCP3 were considered (ns: not significant, *p < 0.05, ****p < 0.0001; Student's *t* test for LepSC, ZygSC and ePacSC; non‐parametric Mann–Whitney *U* test for lPacSC, DipSC).

During meiosis, homologous chromosome synapsis and recombination constitute interdependent processes. Abnormal assembly of the SC central elements in *Sox30* KO spermatocytes (Figure 3B–F) suggests a potential defect in DSB repair. To assess this, the dynamics of DSB formation and repair were evaluated through immunofluorescence co‐staining for SYCP3 and γ‐H2AX. In wild‐type spermatocytes, γ‐H2AX signals exhibited broadly distributed throughout the DSB sites during the leptotene/zygotene stages (Figure 4A). By pachytene/diplotene, these signals became predominantly confined to the XY body (associated with meiotic sex chromosome inactivation), which indicates efficient repair of autosomal DSBs (Figure 4A). Conversely, pachytene/diplotene spermatocytes of *Sox30* KO mice displayed sustained γ‐H2AX staining across autosomal regions, which indicates extensive DSB repair failure (Figure 4A,B).

Efficient DSB repair is essential for CO formation, which mediates genetic exchange between homologous chromosomes and ensures their proper segregation during meiosis. The persistence of unrepaired DSBs in *Sox30* KO spermatocytes suggested potential defects in CO formation. To quantify COs, we performed co‐immunostaining of SYCP3 and MLH1 in pachytene cells, as MLH1 labels mature recombination nodules (Figure 4C). Compared with WT controls, Sox30 KO mice exhibited a significant reduction in the number of MLH1 foci per cell (mean foci per cell: 16 vs. 21; *p* < 0.001), indicating impaired CO formation (Figure 4D). To cytologically validate this crossover defect at a later meiotic stage, we analysed chromosome spreads at metaphase I. This analysis revealed the presence of univalent chromosomes in SOX30 KO spermatocytes (Figure S4C), and quantification showed a significantly increased percentage of metaphase I cells containing univalents compared to controls (Figure S4D). However, we noted that the majority of affected cells contained only a small number of univalents (typically 2), and cells with a higher number (e.g., 8) were rare. This distribution of univalent numbers per cell does not show a direct, one‐to‐one correlation with the average reduction in MLH1 foci, a discrepancy which may reflect asynchronous progression or loss of MLH1 foci at pachytene, or technical challenges in detecting all foci in the mutant background. Regardless of this distribution, the unequivocal presence of univalents provides direct morphological evidence for the CO formation defect.

### 2.5 SOX30 Deficiency Disrupts the HRR Process

During meiosis, the majority of DSBs are repaired via the HRR, where replication protein A (RPA) complex stabilises single‐stranded DNA ends, while RAD51 and DMC1 form nucleoprotein filaments that mediate homologous pairing and strand invasion, critical steps for successful recombination [30, 31]. To investigate the mechanisms underlying the persistent DSBs and reduced CO formation observed in *Sox30* KO spermatocytes, we analysed the dynamics of key proteins involved in meiotic HRR. In wild‐type spermatocytes, RPA2 foci were abundantly present during the leptotene/zygotene stages but progressively diminished as recombinases assembled (Figure 4E,F). In contrast, *Sox30* KO spermatocytes exhibited a significant increase in RPA2 foci localised along chromosome axes starting from the zygotene stage (Figure 4E,F). Notably, these RPA2 signals remained persistently associated with the chromosomes even at the pachytene and diplotene stages. Similarly, analysis of RAD51, a core component of the recombination machinery, revealed abnormal dynamics in Sox30 KO mice. In wild‐type zygotene spermatocytes, RAD51 foci were abundant but progressively decreased as DSBs were repaired. However, in Sox30 KO spermatocytes, RAD51 foci accumulated abnormally and remained unresolved throughout the pachytene and diplotene stages (Figure S4A,B). These findings suggest that although the initiation of HRR appears intact in the absence of *Sox30*, the repair process fails to proceed to completion. This defect is consistent with the persistence of DSB markers and ultimately contributes to impaired CO formation during meiosis. We next examined the expression of RPA2, RAD51, MLH1 and found that SOX30 deficiency also led to marked dysregulation (Figure S5A–C). This molecular signature, upregulated RPA2 and RAD51 alongside downregulated MLH1, is fully consistent with our immunofluorescence findings corroborating the defects in HR repair and crossover formation.

### 2.6 SOX30 Directly Regulates the Transcription of Central Element Genes of the SC

**FIGURE 5:**
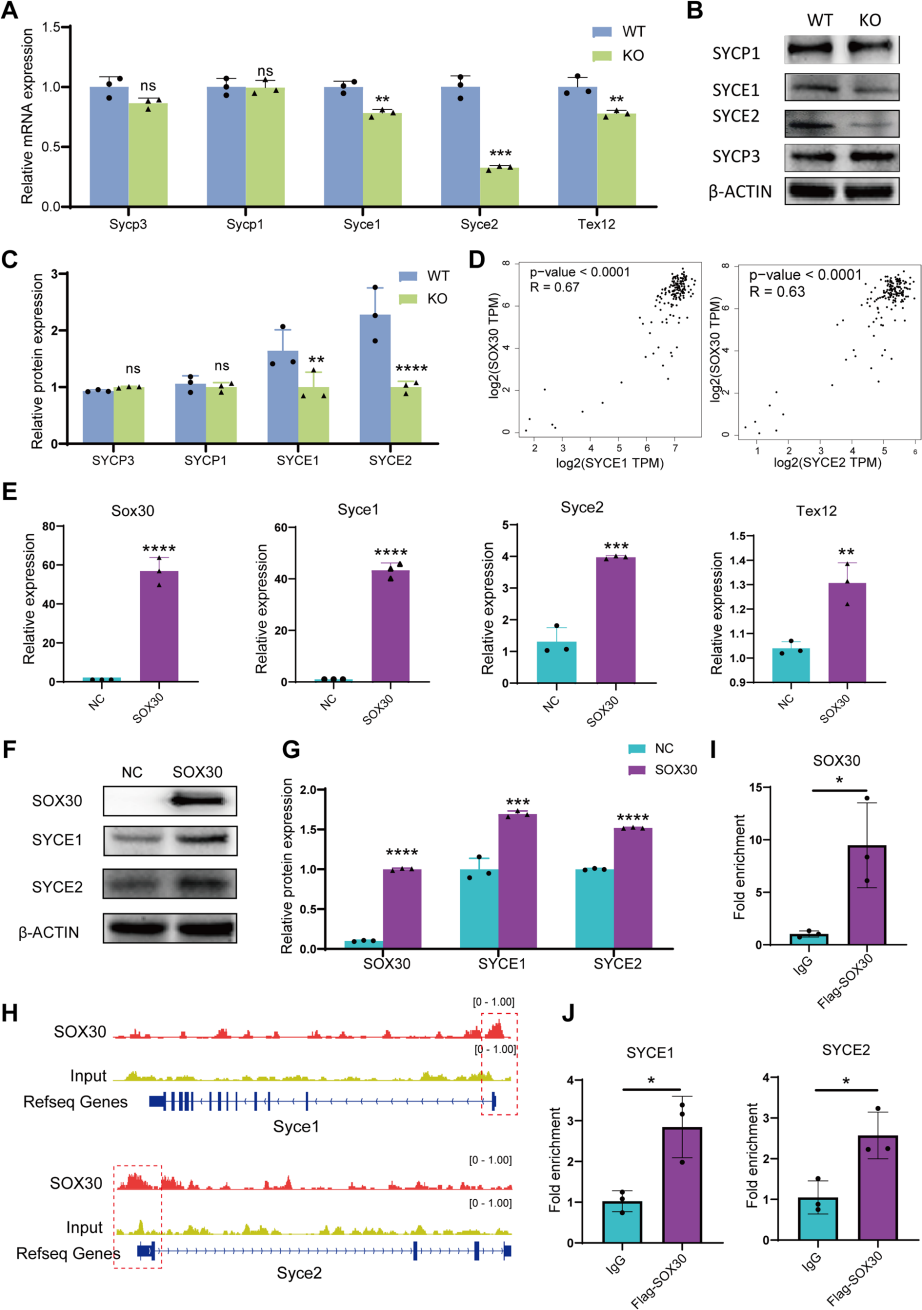
SOX30 directly transcriptionally regulates *SYCE1* and *SYCE2* expression. (A) Bar graph showing mRNA levels of *Sycp3*, *Syce1*, *Syce2* and *Tex12* in testes of WT and KO mice by RT‐qPCR, *n* = 3 for each group. (B) Representative Western blot bands of SYCP3, SYCE1, SYCE2, and SYCP1 protein expression in testes of WT and KO mice. (C) Quantitative analysis of protein expression levels corresponding to (B), *n* = 3 for each group. (D) Correlation scatter plots of *SOX30* with *SYCE1* and *SYCE2* expression in human testis datasets from GEPIA database. (E) qPCR analysis of Sox30, Syce1, Syce2 and Tex12 mRNA levels in GC‐2 cells transfected with either an empty vector (NC, serving as a negative control) or a SOX30 overexpression plasmid (SOX30), *n* = 3 for each group. (F) Representative Western blot bands of SOX30, SYCE1 and SYCE2 protein expression in *SOX30*‐overexpressing GC2 cells. (G) Quantitative analysis of protein expression levels corresponding to (F), *n* = 3 for each group. (H) ChIP‐seq peak profiles at *SYCE1* and *SYCE2* genomic loci in SOX30 chromatin immunoprecipitation assays. (I) ChIP‐qPCR analysis of SOX30 occupancy at (J) *SYCE1* and *SYCE2* promoter regions. *n* = 3 for each group; ns, no significance; *p < 0.05; **p < 0.01; ***p < 0.001; ****p < 0.0001; two‐tailed Student's *t*‐test.

As a canonical transcription factor, SOX30's potential role in regulating SC assembly was investigated using integrated molecular approaches. In *Sox30* KO mouse testes, both qPCR and Western blot analyses revealed significant reductions in the transcript and protein levels of the SC central element genes SYCE1 and SYCE2, and *Tex12* transcript levels were also decreased, whereas its protein expression could not be assessed due to antibody limitations (Figure 5A–C). In contrast, no significant changes were observed in the expression of the SC lateral element SYCP3 and SYCP1 (Figure 5A–C). These findings suggest that SOX30 specifically regulates the expression of SYCE1, SYCE2, and possibly TEX12. To validate this regulatory relationship, SOX30 was overexpressed in GC2 spermatogenic cells, resulting in concurrent upregulation of SYCE1, SYCE2, and TEX12 at both mRNA and protein levels (Figure 5E–G), thereby recapitulating the regulatory phenotype observed in the animal models. Further support for these findings came from human testicular expression data in the GEPIA database [32], which showed strong positive correlations between SOX30 and SYCE1 (Pearson's *r* = 0.67) as well as SYCE2 (Pearson's *r* = 0.63) (Figure 5D).

Mechanistic insights were obtained by analysing published SOX30 chromatin immunoprecipitation sequencing (ChIP‐seq) data (GSE107644) [33], which revealed significant binding peaks within the promoter regions of *Syce1* and *Syce2* (Figure 5H). However, no clear binding peaks were detected in the promoter region of *Tex12*. To confirm these interactions, chromatin immunoprecipitation coupled with quantitative PCR (ChIP‐qPCR) was performed to validate the binding of SOX30 to the promoter regions of *Syce1* and *Syce2*. The results revealed significant enrichment of SOX30 at its endogenous promoter region (Figure 5I), which is consistent with previous reports [16] and served as a positive control. Consistent with our findings in ChIP‐seq data, the core promoter regions of *Syce1* and *Syce2* were significantly enriched by SOX30 (Figure 5J). These results establish SOX30 as a direct transcriptional activator of *Syce1* and *Syce2*, providing a molecular basis underlying the SC assembly defects observed in *Sox30* KO mice.

### 2.7 Reactivation of SOX30 Reverses Synapsis and Recombination Defects

**FIGURE 6:**
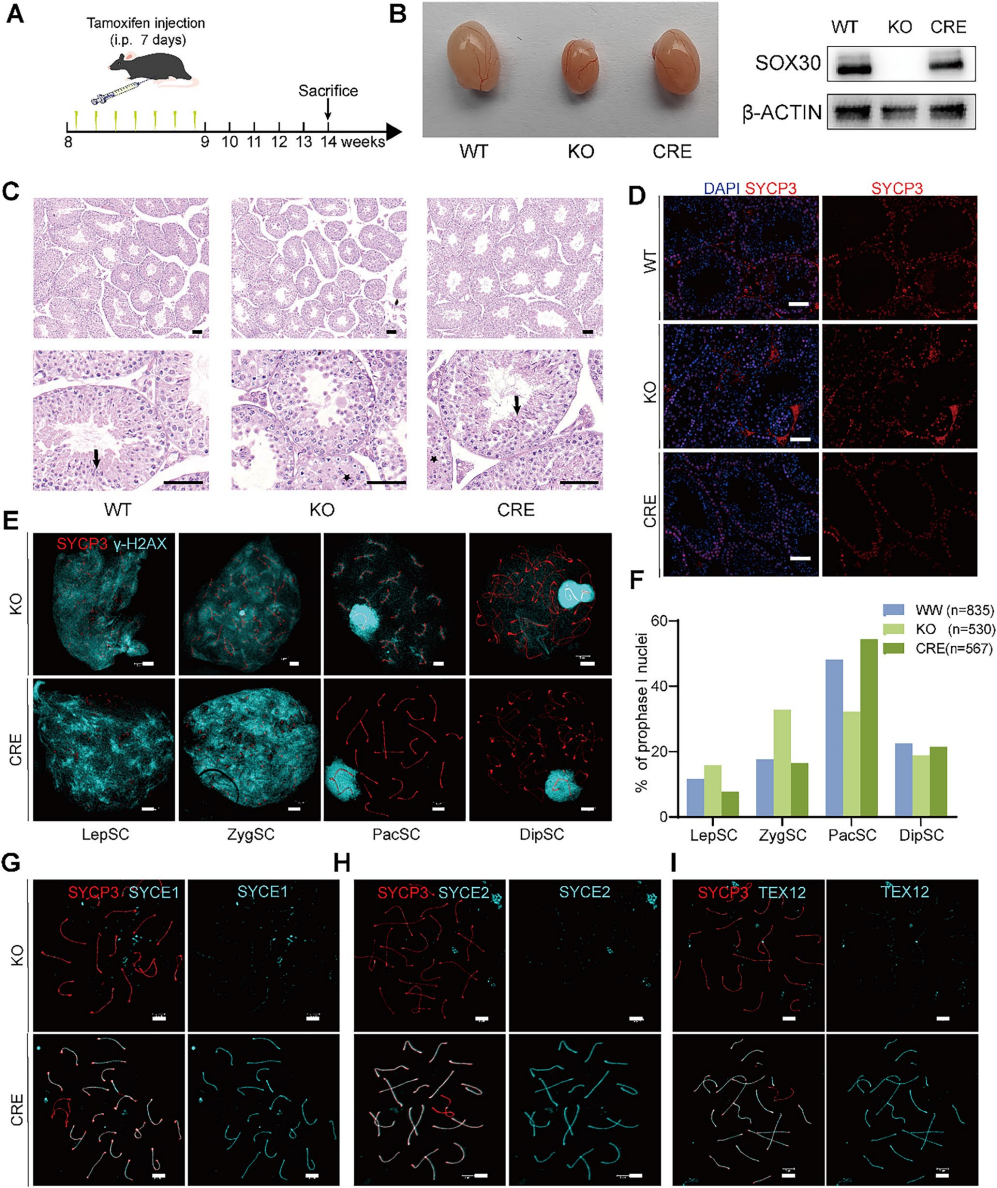
*SOX30* reactivation rescues meiotic arrest. (A) Schematic diagram of *SOX30* expression rescue via intraperitoneal tamoxifen injection. (B) Representative gross morphology of testis size of WT, KO and *SOX30* reactivation mice (CRE). Successful re‐expression of SOX30 in the CRE group is confirmed by western blot analysis. (C) H&E staining of testicular paraffin sections of WT, KO and CRE. Scale bar: 100 μm. (D) SYCP3 immunofluorescence in testicular tissues of WT, KO and CRE mice. Scale bar: 100 μm. (E) Co‐immunofluorescence stain of SYCP3 (red) and γ‐H2AX (cyan) in spermatocyte chromosome spreads of KO and CRE mice. Scale bar: 3 μm. (F) Bar graph showing proportions of spermatocyte subtypes of KO and CRE mice. (G–I) Co‐immunofluorescence stain of SYCP3 (red) and SYCE1, SYCE2, TEX12 (cyan) in spermatocyte chromosome spreads of KO and CRE mice. Scale bar: 3 μm.

To assess the reversibility of SOX30 function, we performed SOX30 reactivation induced by tamoxifen in 8‐week‐old Sox30^flox/flox^ Cre^+^ mice (Figure 6A). Following seven consecutive days of intraperitoneal tamoxifen injections, we observed that testis size had nearly returned to that of WT mice 5 weeks post‐injection (Figure 6B). Western blot analysis confirmed the robust re‐expression of SOX30 protein in the testis of this rescue model (Figure 6B), demonstrating the efficacy of the genetic rescue. The apparent difference in background or non‐specific bands between Figure 1B and Figure 6B is attributable to normal experimental variations, but crucially, both blots consistently show the absence of the specific SOX30 band in the constitutive KO and its successful restoration upon reactivation. Histological analysis showed the presence of mature sperm in the seminiferous tubules of reactivated mice (Figure 6C), although approximately 10% of the seminiferous tubules still contained multinucleated giant cells resembling those observed in *Sox30* KO mice (Figure 6C). Immunofluorescence staining for SYCP3 showed that spermatocyte density and their layered distribution within the seminiferous epithelium were restored to wild‐type patterns (Figure 6D). Chromosome spreading analyses further confirmed no significant difference in the proportion of spermatocytes between reactivated and WT mice (Figure 6E,F), indicating successful rescue of meiotic arrest.

In SOX30‐reactivating spermatocytes, the central elements of SC (SYCE1, SYCE2, TEX12) restored their linear localization along the chromosome axis, structurally reflecting their distribution in WT‐type mouse spermatocytes (Figure 6G–I). In addition, the number of RPA2 foci in zygotic spermatocytes (Figure S6A,B) and the number of MLH1 foci in pachytene spermatocytes (Figure S6C,D) were significantly restored to a level comparable to that of WT mice. Consistently, the mRNA and protein levels of these critical SC and HRR factors were also restored upon SOX30 re‐expression (Figure S7A–C). These results suggest that reactivation of SOX30 effectively rescues meiotic arrest by restoring correct SC assembly and HRR levels.

### 2.8 SOX30 Is a Key Transcription Factor Involved in Regulation of SC and HRR in the Transcriptional Profile of NOA

**FIGURE 7:**
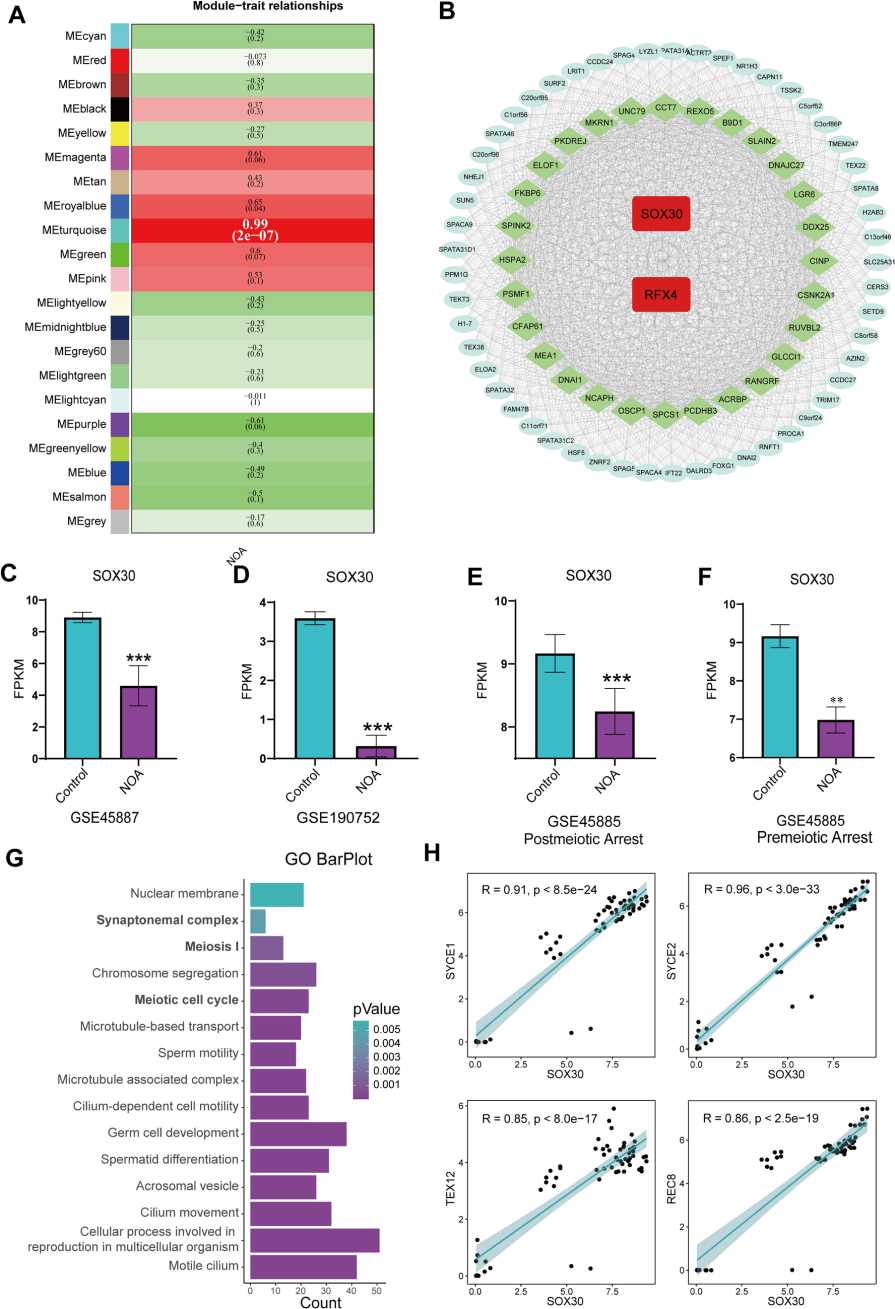
SOX30 is a core transcriptional regulator of SC and HRR in non‐obstructive azoospermia (NOA). (A) Heatmap of module‐trait correlations from WGCNA analysis of NOA testicular transcriptomes (GSE216907). The turquoise module showed the strongest positive correlation with NOA phenotypes (Pearson's *r* = 0.99, *p* < 0.001). (B) Gene regulation network of hub genes (|KME| > 0.98) within the turquoise module, constructed using Cytoscape. Node size reflects connectivity. SOX30 (red) and RFX4 (red) were identified as central transcription factors. (C, D) Bar plots showing significant downregulation of *SOX30* in NOA patients compared to normal controls in datasets GSE45887 (C) and GSE190752 (D). (E, F) SOX30 expression in post‐meiotic arrest (E) and pre‐meiotic arrest (F) NOA subtypes (GSE45885). (**p < 0.01; ***p < 0.001; two‐tailed Student's *t*‐test). (G) GO enrichment analysis of SOX30 co‐expressed genes in NOS testicular transcriptomes. (H) Correlation scatter plot of SOX30 expression levels with SYCE1, SYCE2, TEX12 and REC8 in testicular transcriptome data from NOA patients.

NOA is typically caused by genetic alterations or mutations in critical genes involved in spermatogenesis. Disruption of any of these genes can result in NOA, often accompanied by extensive transcriptional and histological abnormalities. To identify core genes associated with transcriptional dysregulation in NOA, we analysed transcriptomic data from testicular tissues of 8 NOA patients and 2 normal controls (GEO: GSE216907) [34] using Weighted Gene Co‐expression Network Analysis (WGCNA) [35]. This analysis identified 21 co‐expression modules (Figure 7A), among which the turquoise module exhibited the strongest positive correlation with NOA pathogenesis (*R* = 0.99, *p* < 0.001), indicating that it contains the core gene set underlying NOA‐associated transcriptional changes (Figure S8A–C).

Hub genes within this module were selected based on module eigengene‐based connectivity (KME), applying an absolute KME threshold > 0.98. This approach identified 171 hub genes. Using Cytoscape software [36], we constructed a gene regulatory network and identified SOX30 and RFX4 as central transcription factors within this network (Figure 7B), suggesting their pivotal roles in NOA pathogenesis. Literature review revealed that RFX4, like its homologue RFX2, regulates meiosis and spermatogenesis by forming homo or heterodimers that bind to X‐box motifs in the H1T promoter, thereby initiating transcription [37, 38, 39, 40]. Intriguingly, SOX30 has been shown to bind to the RFX2 promoter and regulate its expression [16], supporting its role as a key upstream regulator. These findings establish SOX30 as a core transcriptional regulator in NOA‐associated transcriptional dysregulation. Consistently, SOX30 was found to be downregulated across multiple NOA datasets (Figure 7C,D), including cases involving both pre‐meiotic (Figure 7E) and post‐meiotic arrest (Figure 7F). Functional enrichment analysis of SOX30 co‐expressed genes in testicular transcriptomic data from NOA patients similarly showed significant enrichment for terms related to meiosis and synaptonemal complex organisation (Figure 7G). Additionally, transcriptional correlation analysis of SOX30 with SC formation and HRR genes across NOA and normal testicular transcriptomic datasets revealed strong positive correlations (Figure S9). Moreover, scatter plot analyses further confirmed strong co‐expression relationships between SOX30 and key genes (SYCE1, SYCE2, TEX12, REC8), consistent with the regulatory patterns observed in our mouse models (Figure 7H). These results establish SOX30 as a core of transcriptional network of meiosis, which validates its functional role in human SC assembly and HRR.

## 3 Discussion

**FIGURE 8:**
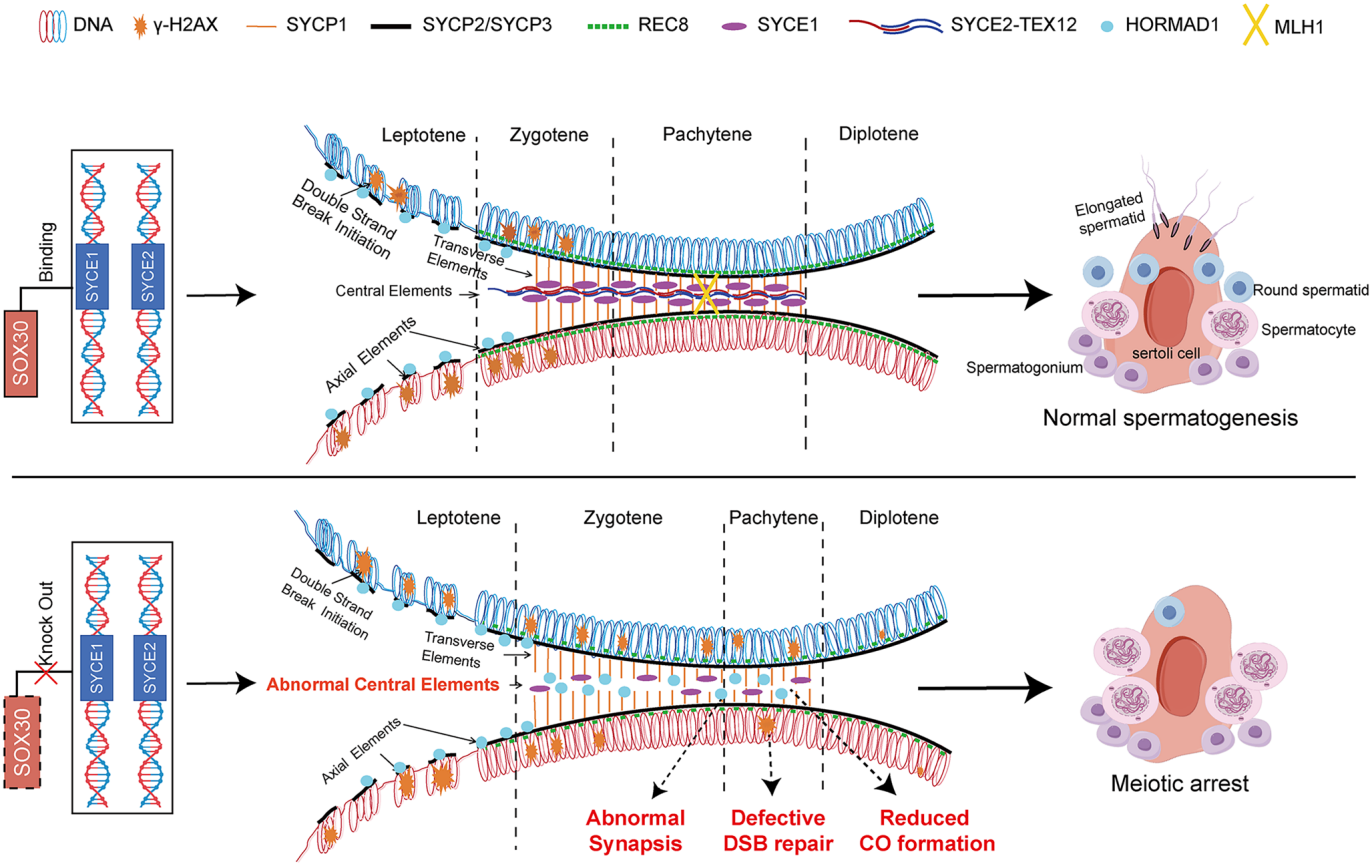
Working model of the transcriptional regulation of SOX30 during male meiosis. The transcription factor SOX30 directly binds promoter regions of SYCE1 and SYCE2 to mediate their transcriptional activation, thereby enabling proper assembly of central elements within the synaptonemal complex. Structural destabilisation of the synaptonemal complex in Sox30 KO spermatocytes triggers synaptic discontinuity, impairs homologous recombination repair, and reduces MLH1‐marked crossover formation leading to meiotic progression arrest at the zygotene.

Meiosis is a specialised form of cell division that underpins gametogenesis in sexually reproducing organisms [36]. The successful meiosis depends not only on the coordinated regulation of complex gene networks but also on the maintenance of stable structural frameworks. Among these, the assembly and stabilisation of the SC provide the essential structural platform for homologous chromosome synapsis and recombination, which ensure the orderly progression of meiotic divisions [2, 3]. Although previous studies have identified several transcription factors, such as MEIOSIN and MYBL1, as key regulators of meiotic gene expression [10, 11], the complete set of transcriptional regulators governing this process remains incompletely understood. Our research identifies SOX30 as a key transcription factor in male meiosis. SOX30 directly regulates the expression of SC genes *Syce1* and *Syce2* via binding to their promoters, which is essential for SC assembly and HRR. SOX30 knockout disrupts the localization of SYCE1/SYCE2/TEX12, leading to incomplete synapsis and impaired HRR, ultimately resulting in meiotic arrest (Figure 8).

Previous studies have demonstrated that SOX30 is a key regulator of spermiogenesis [15, 16, 20]. Sox30‐null mice exhibit striking testicular pathologies, including markedly reduced testis size and weight, complete sterility, and a total absence of spermatozoa, and these phenotypes are rescued by re‐activation of SOX30 [23]. While the essential role of SOX30 in spermatogenesis is now well‐documented, debate persists regarding the precise stage at which it exerts its primary function. SOX30 has been implicated in both late meiosis and post‐meiotic haploid development [15, 16, 20]. Mechanistically, SOX30 governs the transition from late meiotic gene expression programmes to those required for round spermatid differentiation [20]. Our previous work revealed that spermiogenesis in *Sox30* KO male mice is arrested during meiosis, particularly at the zygotene‐to‐pachytene transition, accompanied by abnormal leydig Cell Proliferation [23]. Furthermore, single‐cell transcriptomic analyses demonstrated that SOX30 predominantly drives the differentiation of primary spermatocytes, and its deficiency leads to arrest at the early phase of meiosis I, with a near‐complete absence of secondary spermatocytes [24]. However, the precise mechanisms through which SOX30 mediates these effects remained elusive. In the present study, we demonstrate that SOX30 is crucial for maintaining spermatocyte function by regulating synapsis and HRR during meiosis. Notably, despite the significant accumulation of spermatocytes in the early testes of *Sox30* KO mice, spermatogenesis can still progress to the round spermatid stage at least transiently, although these round spermatids gradually disappear with age. This observation suggests the existence of complex and potentially redundant transcriptional regulatory networks governing both meiosis and spermatogenesis. Conversely, in females, SOX30 exhibits a transient expression peak in the embryonic ovary at E16.5, coinciding with meiotic prophase, and becomes undetectable postnatally [15, 23]. the distinct regulatory mechanisms of meiosis between sexes, combined with its precise temporal expression, suggest that SOX30 may still contribute to early meiotic events such as axis formation. This potential role requires further investigation.

It is well‐established that compensatory mechanisms often operate within such networks, where multiple transcription factors may converge on common developmental processes. The loss of one factor can thus be partially offset by others with overlapping functions. For example, HSF1 and HSF2 exhibit functional compensation: *Hsf1*‐null male mice display abnormal sperm head morphology but remain fertile [37], whereas *Hsf2*‐null males show only mild defects [38, 39]. However, combined deletion of both genes results in a complete absence of spermatocytes and sperm, leading to infertility [38, 39]. The observation of distinct spermatocyte patterns in gene knockout mice further demonstrates the complex regulatory network during meiosis, and such complex network contributes to the stability and recovery of spermatogenesis. For example, two distinct categories of diplonema were identified in Skp1cKO testes based on the staining patterns of HORMAD1 and HORMAD2 [40]. In Redic1 KI mice, although 12.98% of pachytene spermatocytes exhibited fully synapsed chromosomes, the remaining 87.12% displayed various degrees of synaptic abnormalities [41]. Additionally, three types of zygSC‐LCs (zygSC‐L1, 46%; zygSC‐L2, 28%; zygSC‐L3, 26%) were identified in Bend2 KO testes [42]. Similarly, the variable staining patterns of SYCE2 and TEX12 observed in *Sox30* KO spermatocytes suggest the involvement of alternative, compensatory transcription factors. However, this compensatory mechanism becomes insufficient to sustain meiotic progression, which leads to the age‐dependent loss of round spermatids and the persistent spermatocyte phenotypes in Sox30 KO mice. This phenotype confirms the essential role of SOX30 in regulating meiotic progression.

Meiosis is orchestrated by a complex network of transcription factors, including *MEIOSIN*, *MYBL1*, *STRA8*, *E2F1* and *SOHLH1*, which collectively ensure the proper progression of this critical developmental process. In our previous studies, we also found that SOX30 can transcriptionally regulate the expression of STRA8, a key transcription factor in the testis. It indicates that SOX30 plays a relatively upstream role in the transcriptional regulatory network of meiosis. Besides, when exploring genes potentially regulated by SOX30, we identified binding sites of SOX30 in the promoter regions of MYBL1 and MEIOSIN, two key meiotic transcription factors. However, the regulatory relationship between SOX30 and these targets requires further experimental validation.

Furthermore, SOX30 emerged as a core hub gene in NOA patient co‐expression networks. Promoter hypermethylation induced SOX30 downregulation is significantly linked to spermatogenic failure [22]. It is worth noting that the expression of SOX30 in NOA testis tissue with premeiotic arrest is significantly lower than that in NOA testis tissue with postmeiotic arrest, which confirms the high expression of SOX30 in the meiosis stage and shows the important regulatory role of SOX30 in the meiosis process. These findings establish SOX30 as both a diagnostic biomarker for NOA and a clinically therapeutic target to male infertility.

By identifying SOX30 as a transcription factor that links gene regulation to the structural and functional integrity of the SC and HRR, our study uncovers a previously unrecognised regulatory mechanism in male meiosis. These findings not only deepen our understanding of the molecular processes governing SC assembly and HRR fidelity, but also provide novel insights for developing SOX30‐targeted strategies to address male infertility.

## 4 Materials and Methods

### 4.1 Generation of Sox30 Gene Knockout Mice

The *Sox30* KO mouse strain was previously established as described [23]. Briefly, a targeting vector containing homology arms (derived from BAC vector) was constructed by inserting a LoxP‐splice acceptor (SA)‐IRES‐GFP‐Neo‐STOP‐polyA‐LoxP cassette between exons 1 and 2 of the *Sox30* locus (Figure 3A). The targeting construct was validated by PCR, enzyme digestion, and Sanger sequencing and was electroporated into C57BL/6‐derived embryonic stem (ES) cells. Positive ES clones were selected using G418 (200 μg/mL) and ganciclovir (2 μM) dual selection. Validated clones were microinjected to generate chimeric founders. Germline transmission was achieved by breeding chimeras with wild‐type C57BL/6 mice, yielding *Sox30* ^flox/−^ heterozygotes. Intercrossing of heterozygotes produced homozygous *Sox30* ^flox/flox^ (KO) mice.

For inducible *SOX30* reactivation, *Sox30* ^flox/−^ mice were crossed with B6.Cg‐Tg(CAG‐Cre/Esr1)5Amc/JNju tool mice (Jackson Laboratory, stock #004682) expressing tamoxifen‐inducible Cre recombinase. Double‐positive *Sox30* ^flox/flox^ (KO); Cre^+^ offspring were identified by PCR genotyping (primers were described previously) [23]. Tamoxifen‐inducible Cre‐mediated recombination mediates precise excision of the loxP‐flanked STOP cassette in double‐positive (*Sox30* ^flox/flox^; Cre^+^) offspring, thereby restoring endogenous *SOX30* transcriptional activity.

Mice were housed under specific pathogen‐free (SPF) conditions at 22°C ± 2°C with a 12‐h light/dark cycle and provided ad libitum access to standard chow and water. All experiments were carried out with the permission of the Institutional Animal Care and Use Committee of Army Medical University (Approval number: AMUWEC20211007).

### 4.2 Tamoxifen‐Induced Gene Reactivation in CRE‐Positive Knockout Mice

Tamoxifen (HY‐13757A, MCE) was dissolved in corn oil (ST2308, Beyotime) to a final concentration of 20 mg/mL. Eight‐week‐old *Sox30* ^flox/flox^; Cre^+^ male mice received intraperitoneal injections (75 mg/kg) once daily for 7 consecutive days. The mice of the control cohort were treated with corn oil alone. Mice were euthanised 4 weeks post‐injection for tissue collection. Testes were harvested and processed for histological analysis (H&E staining) and molecular validation (qPCR/Western blot).

### 4.3 Histological Analysis

Testis were detached and immediately fixed in 4% paraformaldehyde (PFA) for 24 h at 4°C. Fixed tissues were washed three times with PBS to remove residual PFA and dehydrated through a graded ethanol series (70%, 80%, 90%, 95% and 100%) for 1 h per concentration. Subsequently, tissues were cleared in xylene and embedded in paraffin. Paraffin‐embedded tissues were sectioned at 5‐μm thickness using a rotary microtome and mounted onto poly‐l‐lysine‐coated glass slides. Following deparaffinisation in xylene and rehydration through a graded ethanol series, the sections were stained with haematoxylin and eosin (H&E) according to standard histological protocols for microscopic evaluation of testicular architecture.

### 4.4 Immunofluorescence Staining

Paraffin‐embedded sections (5‐μm thickness) underwent standard deparaffinisation through xylene immersion and rehydration in a graded ethanol series. Antigen retrieval was performed by heating slides in 10 mM sodium citrate buffer (pH 6.0) at 95°C for 20 min using a controlled water bath. To minimise non‐specific binding, sections were blocked for 1 h at room temperature with a solution containing 5% bovine serum albumin (BSA; B265994, aladding), and 0.05% Triton X‐100 (ST1723, Beyotime) in PBS (pH 7.4).

Primary antibodies (detailed in Table S1) were diluted in blocking buffer and applied to sections overnight at 4°C in a humidified chamber. After 10‐min washes with PBS containing 0.05% Tween‐20 (PBST) for three times, species‐matched Alexa Fluor‐conjugated secondary antibodies (detailed in Table S1) were incubated at 37°C for 1 h protected from light. Then the sections were washed with PBST for 10 min three times.

For nuclear counterstaining and mounting, sections were coverslipped with Antifade Mountant containing DAPI (BL739B, biosharp) and stored at 4°C in the dark to preserve fluorescence signal. Imaging was conducted using: Confocal microscopy: Leica SP8 laser scanning confocal system equipped with a 40×/1.40 NA oil immersion objective. Fluorescence signals were acquired sequentially using 405 nm (DAPI), 488 nm (Alexa Fluor 488) and 594 nm (Alexa Fluor 594) laser lines. Z‐stack images were captured at 1‐μm intervals and processed with Leica LAS X software (v3.7.4).

### 4.5 Spermatocyte Chromosome Spreading and Immunofluorescence Staining

Meiotic chromosome spreads were prepared from spermatocytes using a dry‐down method [43]. Testicular tissues were harvested from 2 to 4 months old mice, and seminiferous tubules were isolated in PBS (pH 7.4) and incubated in hypotonic extraction buffer (30 mM Tris–HCl, 50 mM sucrose, 17 mM sodium citrate, 5 mM EDTA, 0.5 mM DTT, pH 8.2) for 45 min to induce cell swelling. Then the swelling germ cells were squeezed out in 100 mM sucrose solution (pH 8.2) and spread on slides with 1% PFA containing 0.15% Triton X‐100. The slides were incubated in a humidity chamber at room temperature for 2.5 h and air dried. Then the slides were stored at −80°C for immunofluorescence stain. Following equilibration to room temperature, frozen chromosome spread slides were subjected to PBST with gentle orbital shaking (100 rpm) for 5 min. Non‐specific binding was blocked by incubating slides in 5% non‐fat dry milk (PS112, Epizyme) diluted in PBST for 2 h at room temperature. Primary antibodies (see Table S1) were applied to the slides and incubated overnight at 4°C. After three stringent PBST washes, species‐matched Alexa Fluor‐conjugated secondary antibodies were incubated for 2 h at room temperature protected from light. Following secondary antibody incubation, slides were washed in PBST, and coverslipped with VECTASHIELD Antifade Mounting Medium (Vector Laboratories, #H‐1000). Imaging was conducted using: Confocal microscopy: Leica SP8 laser scanning confocal system equipped with a 100×/1.40 NA oil immersion objective. Images are processed using Lighting mode in LAS X software after acquisition for high‐resolution imaging.

### 4.6 GC2 Cell Culture and Adenoviral‐Mediated Sox30 Overexpression

GC2 spermatocyte‐derived cells (Cell Bank of Chinese Academy of Science) were maintained in DMEM medium (11,965,092, Gibco) supplemented with 10% fetal bovine serum (FBS; FB25015, Clark) and 1% penicillin/streptomycin at 37°C under 5% CO_2_.

Adenoviral vector construction was finished by Genomeditech: The murine *Sox30* coding sequence (NCBI RefSeq: NM_173384.2) was cloned into a lentiviral vector (PGMLV‐CMV) containing an N‐terminal FLAG tag and puromycin resistance. GC2 cells were infected with lentivirus (MOI = 20) in DMEM supplemented with 5 μg/mL polybrene for 24 h. Stable pools were selected using 2 μg/mL puromycin for 72 h. The overexpression efficiency of *Sox30* was quantitatively validated through western blotting and quantitative PCR analyses.

### 4.7 Quantitative Reverse Transcription Polymerase Chain Reaction (RT‐qPCR)

Total RNA was isolated from testicular tissues/cells using TRIZOL reagent (R401, Novizan), followed by chloroform extraction and isopropanol precipitation. RNA purity (A260/A280 = 1.8–2.1) and concentration were quantified via NanoDrop One (Thermo Fisher). cDNA synthesis was performed with 1 μg RNA using the ABScript III RT Master Mix (RK20429, abclonal). qPCR reactions (SYBR Green Fast qPCR Mix; RK21203 abclonal) were run on a QuantStudio 3 system (Thermo Fisher) with gene‐specific primers (Table S2). Cycling conditions: 95°C/3 min; 40 cycles of 95°C/5 s, 60°C/30 s. Data were normalised to *Gapdh* using the 2^−ΔΔCt^ method. Primer specificity was confirmed by melt curve analysis.

### 4.8 Western Blotting

Testes from adult C57BL/6 mice (8–12 weeks old) were homogenised in ice‐cold RIPA lysis buffer (50 mM Tris–HCl pH 7.5, 150 mM NaCl, 2.5 mM EDTA, 0.5% Triton X‐100) supplemented with protease inhibitor cocktail (P1045, Beyotime). Lysates were sonicated with an ultrasonic processor equipped with a 3‐mm probe (3 × 10 s pulses at 40% amplitude with 30 s cooling intervals), then centrifuged at 15,000 × *g* for 15 min at 4°C.

Protein concentrations were determined by BCA assay (P0010, Beyotime). Equal amounts of protein were denatured in loading buffer (LT101, Epizyme Biotech) at 95°C for 10 min, resolved on SDS‐PAGE gels (PG610, Epizyme Biotech), and transferred to PVDF membranes (0.45 μm pore size; IPVH00010, Merck) using a submerged transfer system (Bio‐Rad Trans‐Blot Cell). Membranes were blocked with 5% non‐fat dry milk (PS112, Epizyme Biotech) in TBST (20 mM Tris–HCl pH 7.6, 150 mM NaCl, 0.1% Tween‐20) for 1 h at room temperature, followed by incubation with primary antibodies (diluted in blocking buffer) overnight at 4°C. After three 10‐min TBST washes, membranes were incubated with secondary antibodies for 2 h at room temperature. Chemiluminescent signals were developed using SuperSignal West Dura (34,075, Thermofisher) and captured with a Fusion FX7 system (Vilber, Korea). Antibody specifications are detailed in the Table S2.

### 4.9 Chromatin Immunoprecipitation‐qPCR

Sonication ChIP Kit was used in this section (RK20258, Abclone). GC2 cells overexpressing FLAG‐tagged SOX30 were crosslinked with 1% formaldehyde for 10 min on ice, followed by glycine quenching. Chromatin was sonicated to 200–500 bp fragments using an ultrasonic processor equipped (30 cycles of 30 s ON/30 s OFF). Pre‐cleared chromatin lysates (100 μg) were incubated overnight at 4°C with anti‐FLAG antibody (#M20008, Abmart) or IgG control (#AC005, Abmart), followed by 2 h incubation with MCE Protein A/G Magnetic Beads (#HY‐K0202, MedChemExpress). Beads were washed sequentially with low‐salt (0.1% SDS, 1% Triton X‐100, 2 mM EDTA), high‐salt (500 mM NaCl), and LiCl buffers. Reversed crosslinked DNA (65°C overnight) was purified using the DNA/RNA Isolation Kit (R6731, Promega) and eluted in 50 μL nuclease‐free water. Enrichment of target loci was quantified via SYBR Green qPCR (Abclonal) on a QuantStudio 3 system. Primers used for qPCR are listed in Table S3. Fold enrichment was calculated as 2^−ΔΔC^ normalised to Input DNA and IgG controls.

### 4.10 Single‐Cell Data Analysis

Raw sequencing reads were aligned to the mouse reference genome (GRCm38/mm10 assembly, UCSC version) utilising Cell Ranger software (v7.0.1), with default splice‐aware parameters. Unique molecular identifiers (UMI) demultiplexing and cell barcode identification were performed to generate cell genes count matrix.

Raw gene expression matrices generated by Cell Ranger (v7.1.0) were imported into Seurat (v4.3.0) for downstream analysis [44]. The WT and KO experimental groups were merged using the merge function, retaining cellular barcodes with ≥ 1000 detected genes and < 10% mitochondrial reads. Counts were log‐normalised and 3000 highly variable genes (HVGs) were identified using the *vst* method. Principal component analysis (PCA) was conducted on scaled HVGs (*n* = 20 PCs retained). Cells were clustered using the Louvain algorithm (resolution = 0.7). Uniform Manifold Approximation and Projection (UMAP) was employed for 2D visualisation. The proportions of cell types across groups were calculated and compared.

### 4.11 Cell Type Definition

‘Ccnd2’ is predominantly expressed in spermatogonial stem cells, playing a crucial role in mitotic renewal. In contrast, ‘Stra8’ is highly expressed in differentiated spermatogonia, facilitating the transition from mitosis to premeiosis, and acts as a key molecular switch for male germ cells entering meiosis. To identify the zygotene phase, we focused on the expression patterns of ‘Sycp1’, which encodes a component of the SC, and ‘Dmc1’, a homologous recombination repair enzyme. Additionally, we observed significant high‐level expression of ‘Ndufa1’ and ‘Prss50’ at the zygotene stage, suggesting these genes as potential markers for this stage. Our exploration of public databases also identified ‘Tuba3b’, ‘Smim24’, and ‘Spink2’ as being highly expressed in pachytene spermatocytes, which was different from that in diplotene spermatocytes. For diplotene and later‐stage meiotic spermatocytes, genes such as ‘Magi2’, ‘Agbl4’, ‘Lekr1’, and ‘Catsperb’, involved in sperm maturation and other processes, were predominantly transcribed.

### 4.12 Developmental Trajectory Analysis

Single‐cell trajectories were inferred using CytoTRACE (v0.3.3) on the processed Seurat object containing germ cell subsets [45]. Gene counts were normalised via scTransform prior to analysis. The CytoTRACE score, reflecting differentiation potential, was calculated using all expressed genes (nFeature_RNA > 500) with default parameters. Pseudotime values were projected onto UMAP coordinates. Trajectory consistency was validated through bootstrap resampling (*n* = 100 iterations).

### 4.13 Weighted Gene co‐Expression Network Analysis

Transcriptomic datasets from 8 non‐obstructive azoospermia (NOA) patients and 2 normal testes (GSE216907) were retrieved from the GEO database. Co‐expression networks were constructed using the WGCNA R package (v1.72) [35]. A soft threshold power (*β* = 12) was selected to satisfy scale‐free topology. A signed adjacency matrix was converted to topological overlap matrix (TOM) to minimize spurious correlations. Dynamic tree cutting (minClusterSize = 30) identified 21 co‐expression modules. Eigengenes representing module expression patterns were correlated with clinical subtypes (NOA, normal) using Pearson's correlation. Hub genes within selected modules were rigorously identified by calculating module eigengene‐based connectivity (KME) using the moduleEigengenes function in WGCNA. These module genes show strongest co‐expression with module eigengenes. Hub genes were functionally annotated via clusterProfiler R package [46].

### 4.14 Gene Set Enrichment Analysis (GSEA)

Raw RNA‐seq data from *Sox30*‐knockout and wild‐type mouse testes (GSE113073) were retrieved from the GEO database. Differentially expressed genes (DEGs) were identified using DESeq2 (v1.38.3) with thresholds of |log_2_FC| > 1 and adjusted *p* < 0.05. Hallmark gene sets from MSigDB (v2023.1) were analysed using the clusterProfiler R package (v4.8.1). Enrichment scores were calculated with 1000 permutations, and significant pathways were filtered by normalised enrichment score (|NES| > 1).

### 4.15 Heatmap Visualisation

*Z*‐Score normalised expression values were plotted using the pheatmap R package (v1.0.12). Hierarchical clustering (Euclidean distance, complete linkage) was applied to both rows (genes) and columns (samples). Colour gradients were scaled from −2 (blue) to +2 (red) to represent expression deviations.

### 4.16 Statistical Analysis

Data are presented as mean ± SEM. Comparisons between two groups used Student's t‐test (unpaired, two‐tailed); multiple groups were analysed by one‐way ANOVA with Tukey's post hoc test. Correlation analyses employed Pearson/Spearman coefficients. Statistical significance was defined as *p* < 0.05. Analyses were performed in GraphPad Prism (v9.0) or R (v4.3.1).

## Author Contributions

**Kangle Liu:** conceptualization, methodology, software, validation, writing – original draft, visualisation. **Wenfeng Zhang:** conceptualisation, methodology, validation, visualisation. **Xiao Jiang:** conceptualisation, methodology, software, funding acquisition. **Jianping Chen:** conceptualisation, methodology. **Lei Zhu:** visualisation, supervision. **Zhonghao Zhang:** supervision, writing – review and editing. **Jing Gu:** supervision. **Lulu Guo:** supervision. **Lin Ao:** supervision. **Qing Chen:** supervision. **Lei Sun:** supervision. **Yuhan Hu:** supervision. **Xin Wang:** supervision. **Jia Cao:** conceptualisation, investigation, resources, writing – review and editing, project administration. **Fei Han:** conceptualisation, investigation, resources, writing – review and editing, project administration, funding acquisition. **Jinyi Liu:** conceptualisation, methodology, investigation. resources, writing – review and editing, project administration, funding acquisition.

## Funding

This work was supported by the National Natural Science Foundation of China (Grant No. 82173555 and 82073137); and by the Natural Science Foundation of Chongqing (Grant No. cstc2021jcyj‐msxmX0316).

## Conflicts of Interest

The authors declare no conflicts of interest.

## Supporting information


**Figure S1:** Spatiotemporal expression and meiotic regulatoion of SOX30. (A) Co‐immunofluorescence of SOX30 (red) and SYCP3 (axial element, green) in testicular sections. SOX30 specifically localises to zygotene‐stage spermatocytes and subsequent meiotic/postmeiotic cells (arrowheads). Scale bar: 4 μm. (B, C) Violin plots showing temporal expression patterns of SOX30 mRNA in human (B) and mouse (C) spermatogenic cells from the MHA single‐cell transcriptome database. Expression peaks during zygotene‐pachytene stages. (D) Heatmap of differentially expressed meiotic genes in *Sox30* KO vs. WT testes. (E, F) GSEA enrichment plots demonstrating significant suppression of ‘Negative regulation of meiotic nuclear division’ (E, NES = −1.68, FDR = 0.004) and ‘Meiotic cell cycle’ (F, NES = −1.279, FDR = 0.01) pathways in KO testes
**Figure S2:** Single‐cell transcriptomic validation of spermatogenic lineage identity and developmental disruption in SOX30 deficiency. (A) Dot plot of marker genes defining spermatogenic cell types in the MHA single‐cell RNA‐seq atlas
**Figure S3:** SOX30 ensures chromosomal axis integrity through coordinated regulation of synaptonemal and cohesin complexes. (A) SYCE2 (central element, cyan) and SYCP3 (lateral element, red) co‐staining in chromosome spreads of *Sox30* KO pachytene spermatocytes. Two distinct populations emerge: PacSC‐I (178/193, 92.2% of cells) exhibit complete SYCE2 delocalization from chromosomal axes, while PacSC‐II (15/193, 7.8%) retain wild‐type‐like SYCE2/SYCP3 co‐localization. Scale bar: 3 μm. (B) Schematic of physiological REC8 (cohesin complex, cyan) distribution along SYCP3‐marked chromosomal axes in WT spermatocytes. (C) Western blot analysis of testicular REC8 protein levels. β‐actin loading control shown
**Figure S4:** SOX30 deficiency causes defective HRR and reduced crossover formation. (A) SYCP3 (red) and RAD51 (recombination intermediates, cyan) co‐localization in WT and Sox30 KO spermatocytes. Scale bar: 3 μm. (B) Scatterplot quantifying RAD51 foci counts across meiotic stages. X‐axis numbers indicate cells from 3 mice for per stage. (ns: not significant, ***p < 0.001, non‐parametric Mann–Whitney *U* test). (C) Representative metaphase I spreads from control (left) and KO (right) mice, with arrows indicating univalents in the KO cell. (D) The percentage of metaphase I cells with univalents is significantly increased in KO testes (*n* = 3, ****p < 0.0001, non‐parametric Mann–Whitney U test).
**Figure S5:** SOX30 deficiency alters the expression of key HRR factors. (A) qPCR analysis shows mRNA expression changes of Rpa2 and Rad51 (*n* = 3). (B) Representative western blots of indicated HRR proteins. (C) Quantitative analysis of protein levels from (B) (*n* = 3). Data are mean ± SEM. **p < 0.01, ***p < 0.001; ns, not significant (unpaired *t*‐test)
**Figure S6:** SOX30 reactivation rescues recombination repair defects in spermatocytes. (A) SYCP3 (lateral element, red) and RPA2 (recombination intermediate, cyan) co‐staining in *Sox30* KO and inducible Sox30‐rescued (CRE) spermatocytes. Scale bar: 3 μm. (B) Scatterplot comparing RPA2 foci counts across meiotic stages. Numbers below the *x*‐axis labels indicate cells analysed per group (ns: not significant, *p < 0.05, ***p < 0.001, ****p < 0.0001, One‐way ANOVA test). (C) SYCP3 (red) and MLH1 (crossover sites, cyan) co‐localization in *Sox30* KO and CRE spermatocytes. Scale bar: 3 μm. (D) Scatterplot of MLH1 foci counts in pachytene spermatocytes. (ns: not significant, ****p < 0.0001, One‐way ANOVA test)
**Figure S7:** SOX30 reactivation rescues the expression of key meiotic genes. (A) qPCR analysis of synaptonemal complex and HRR gene mRNAs (*n* = 3). (B) Representative western blots of SC and HRR proteins. (C) Quantitative analysis of protein levels from (B) (*n* = 3). *p < 0.05, **p < 0.01, ***p < 0.001; ns, not significant
**Figure S8:** WGCNA identifies SOX30 as a hub gene in co‐expression networks associated with non‐obstructive azoospermia (NOA). (A) Soft threshold power selection for weighted gene co‐expression network construction. A power value of *β* = 12 was chosen based on scale‐free topology fit (*R* ^2^ > 0.9) and mean connectivity criteria. (B) Left: Eigengene adjacency heatmap showing pairwise correlations between co‐expression modules. Right: Cluster dendrogram of genes with dissimilarity cut defining 18 distinct modules. (C) Network heatmap plot depicting topological overlap matrix (TOM) for all genes. Genes within the turquoise module form a densely interconnected subnetwork.
**Figure S9:** Correlation heatmap of SOX30 and synapsis/homologous recombination repair‐related genes in testicular transcriptome data from NOA patients
**Table S1:** Antibodies used in this study
**Table S2:** Primer sequences for qPCR analysis used in this study
**Table S3:** Primer sequences for ChIP‐qPCR analysis

## Data Availability

The datasets used and/or analysed during the current study are available from the corresponding author on reasonable request.
