# Supplementary material for: SOX30 Governs Synaptonemal Complex Assembly and Homologous Recombination in Male Meiosis
Source: Cell Prolif. 2025 Dec 30;59(6):e70158. doi: 10.1111/cpr.70158 (PMC13241807; doi:10.1111/cpr.70158)
Supplement: Supplementary file 1 — Figure S1: Spatiotemporal expression and meiotic regulatoion of SOX30. (A) Co‐immunofluorescence of SOX30 (red) and SYCP3 (axial element, green) in testicular sections. SOX30 specifically localises to zygotene‐stage spermatocytes and subsequent meiotic/postmeiotic cells (arrowheads). Scale bar: 4 μm. (B, C) Violin plots showing temporal expression patterns of SOX30 mRNA in human (B) and mouse (C) spermatogenic cells from the MHA single‐cell transcriptome database. Expression peaks during zygotene‐pachytene stages. (D) Heatmap of differentially expressed meiotic genes in Sox30 KO vs. WT testes. (E, F) GSEA enrichment plots demonstrating significant suppression of ‘Negative regulation of meiotic nuclear division’ (E, NES = −1.68, FDR = 0.004) and ‘Meiotic cell cycle’ (F, NES = −1.279, FDR = 0.01) pathways in KO testes Figure S2: Single‐cell transcriptomic validation of spermatogenic lineage identity and developmental disruption in SOX30 deficiency. (A) Dot plot of marker genes defining spermatogenic cell types in the MHA single‐cell RNA‐seq atlas Figure S3: SOX30 ensures chromosomal axis integrity through coordinated regulation of synaptonemal and cohesin complexes. (A) SYCE2 (central element, cyan) and SYCP3 (lateral element, red) co‐staining in chromosome spreads of Sox30 KO pachytene spermatocytes. Two distinct populations emerge: PacSC‐I (178/193, 92.2% of cells) exhibit complete SYCE2 delocalization from chromosomal axes, while PacSC‐II (15/193, 7.8%) retain wild‐type‐like SYCE2/SYCP3 co‐localization. Scale bar: 3 μm. (B) Schematic of physiological REC8 (cohesin complex, cyan) distribution along SYCP3‐marked chromosomal axes in WT spermatocytes. (C) Western blot analysis of testicular REC8 protein levels. β‐actin loading control shown Figure S4: SOX30 deficiency causes defective HRR and reduced crossover formation. (A) SYCP3 (red) and RAD51 (recombination intermediates, cyan) co‐localization in WT and Sox30 KO spermatocytes. Scale bar: 3 μm. (B) Scatterplot quant [file CPR-59-e70158-s001.docx]

**Supplementary Information**

**Discovery of SOX30 as a Central Regulator of Synaptonemal Complex Assembly and Homologous Recombination in Male Meiosis**

Kangle Liu^1,2,3,^ ^†^, Wenfeng Zhang^1,2,3,^ ^†^, Xiao Jiang^1,2,3,†^, Jianping Chen^1,2,3^, Lei Zhu^1,2,3^, Zhonghao Zhang^1,2,3^, Jing Gu^1,2,3^, Lulu Guo^1,2,3^, Lin Ao^1,2,3^, Qing Chen^1,2,3^, Lei Sun^1,2,3^, Yuhan Hu^1,2,3^, Xin Wang^1,2,3^, Yaxin Liu^1,2,3^, Jia Cao^1,2,3*^, Fei Han^4*^, Jinyi Liu^1,2,3*^

1 Institute of Toxicology, College of Preventive Medicine, Army Medical University, Chongqing 400038, China.

2 State Key Lab of Trauma and Chemical Poisoning, and Key Lab of Medical Protection for Electromagnetic Radiation, Ministry of Education of China, Army Medical University, Chongqing 400038, China.

3 Chongqing Municipal Key Laboratory of Hygiene Toxicology of Higher Education, Chongqing 400038, China.

4 Joint International Research Laboratory of Reproduction and Development of the Ministry of Education, School of Public Health, Chongqing Medical University, Chongqing 400016, China.

† These authors contributed equally to this work.

*** Correspondence:** caojia1962@126.com (Jia Cao); han16897723@163.com, [flyskyhf@cqmu.edu.cn](mailto:flyskyhf@cqmu.edu.cn) (Fei Han); jinyiliu@tmmu.edu.cn , jinyiliutmmu@163.com (Jinyi Liu).

Figs. S1 to S7

Tables S1 to S3


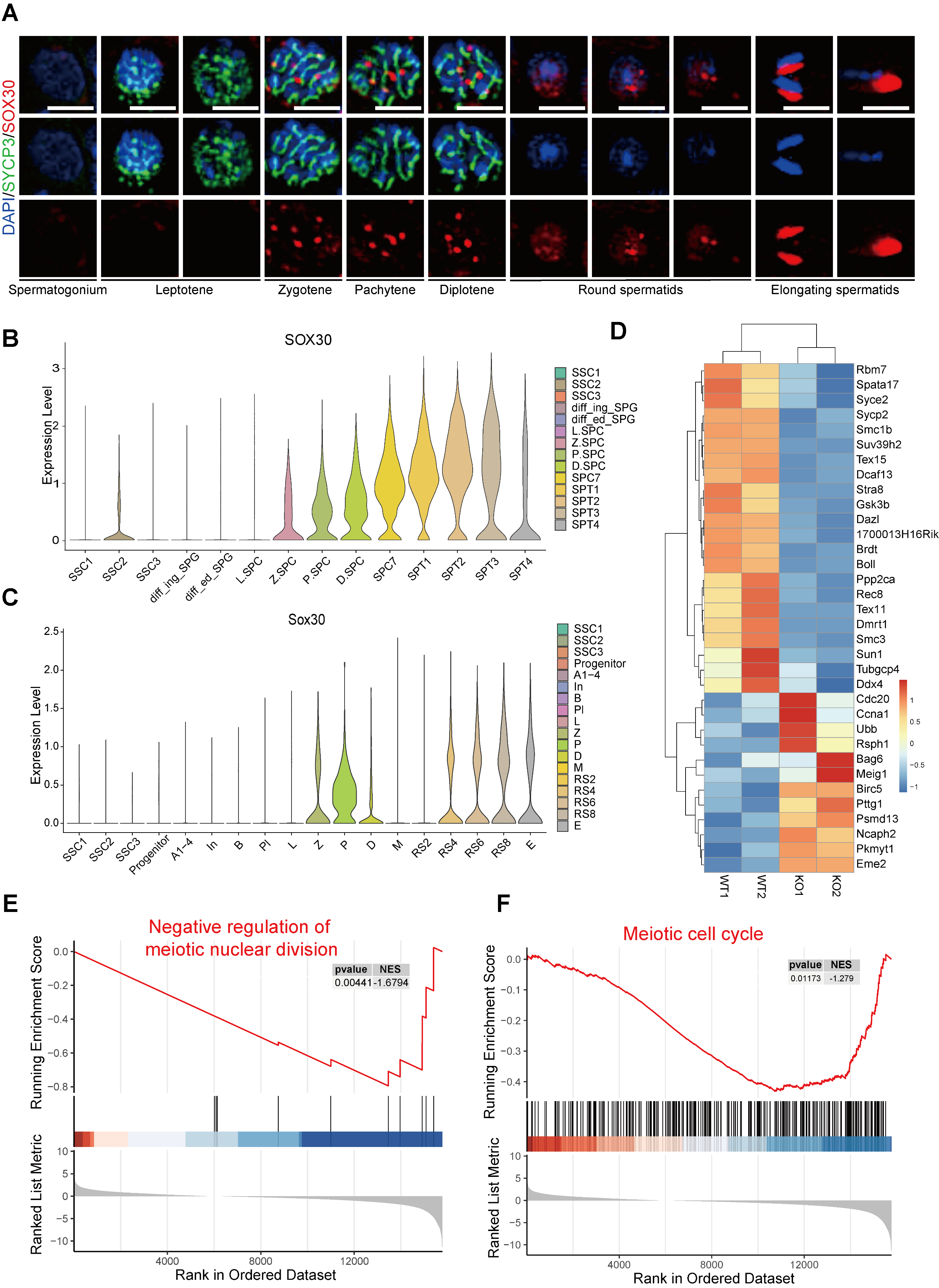
**Figure S1. Spatiotemporal expression and meiotic regulatoion of SOX30.** (A) Co-immunofluorescence of SOX30 (red) and SYCP3 (axial element, green) in testicular sections. SOX30 specifically localizes to zygotene-stage spermatocytes and subsequent meiotic/postmeiotic cells (arrowheads). Scale bar: 4 μm. (B-C) Violin plots showing temporal expression patterns of SOX30 mRNA in human (B) and mouse (C) spermatogenic cells from the MHA single-cell transcriptome database. Expression peaks during zygotene-pachytene stages. (D) Heatmap of differentially expressed meiotic genes in *Sox30* KO vs. WT testes. (E-F) GSEA enrichment plots demonstrating significant suppression of "Negative regulation of meiotic nuclear division" (E, NES = -1.68, FDR = 0.004) and "Meiotic cell cycle" (F, NES = -1.279, FDR = 0.01) pathways in KO testes.


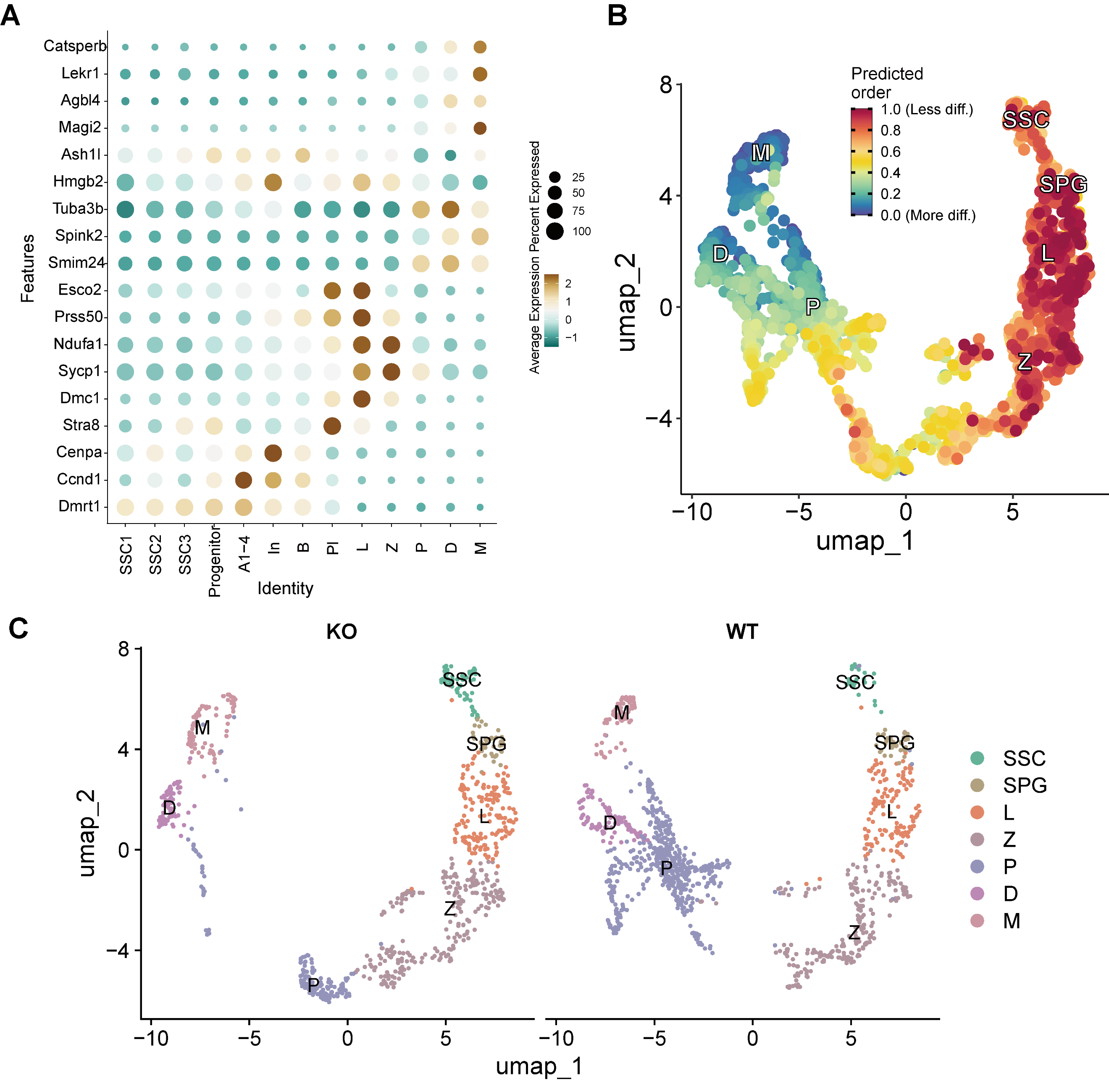


**Figure S2. Single-cell transcriptomic validation of spermatogenic lineage identity and developmental disruption in SOX30 deficiency. (**A) Dot plot of marker genes defining spermatogenic cell types in the MHA single-cell RNA-seq atlas.

(B) Cytoscape-generated pseudotime trajectory heatmap of single cell RNA seq data of Sox30 KO spermatogonia and spermatocytes. (C) UMAP projections of the single-cell data, split by genotype (KO, left; WT, right), showing the distribution of spermatogenic cell populations. SSC: spermatogonial stem cell; SPG: spermatogonial; L: Leptotene; Z: Zygotene; P: Pachytene; D: Diplotene; M: Metaphase


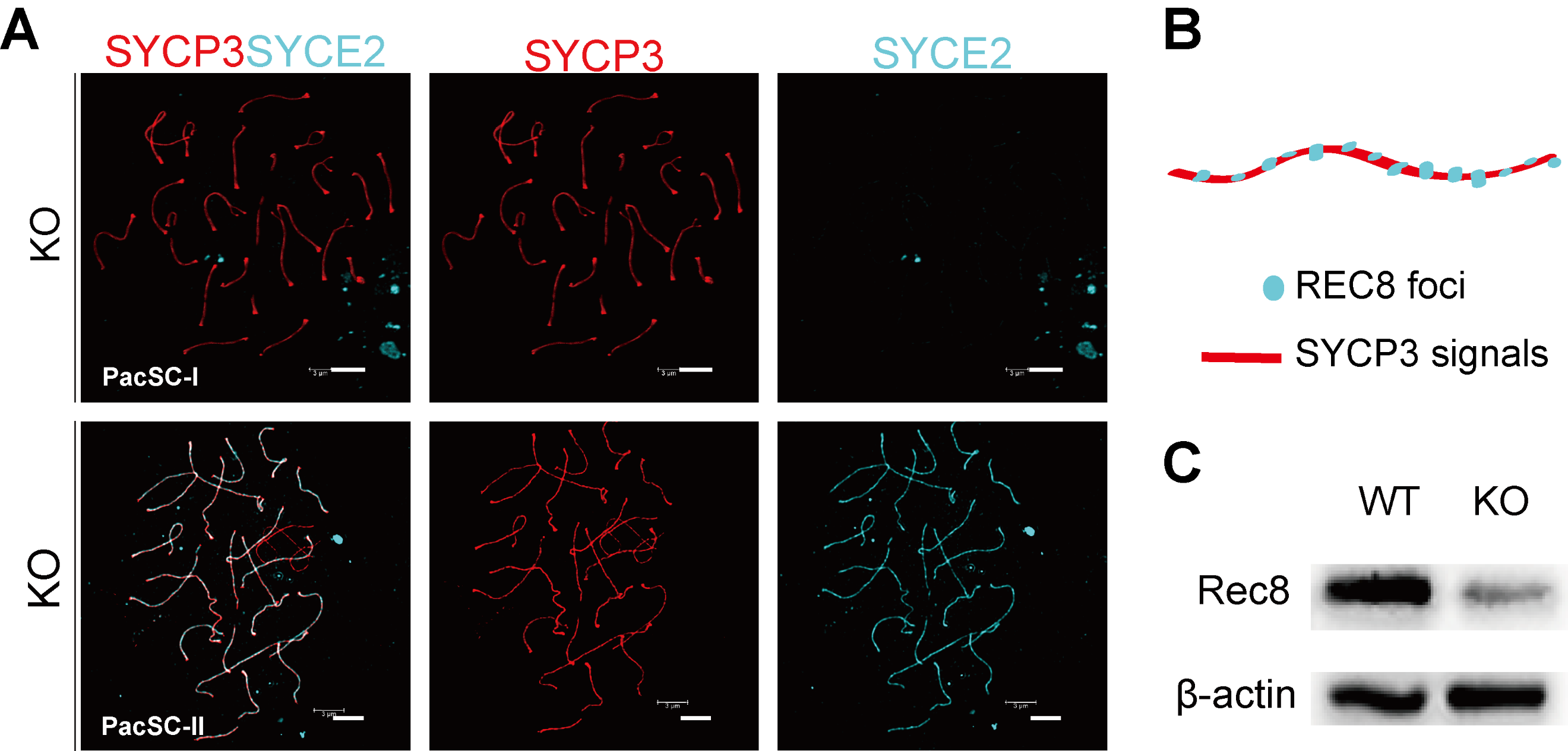


**Figure S3. SOX30 ensures chromosomal axis integrity through coordinated regulation of synaptonemal and cohesin complexes.** (A) SYCE2 (central element, cyan) and SYCP3 (lateral element, red) co-staining in chromosome spreads of *Sox30* KO pachytene spermatocytes. Two distinct populations emerge: PacSC-I (178/193, 92.2% of cells) exhibit complete SYCE2 delocalization from chromosomal axes, while PacSC-II (15/193, 7.8%) retain wild-type-like SYCE2/SYCP3 co-localization. Scale bar: 3 μm. (B) Schematic of physiological REC8 (cohesin complex, cyan) distribution along SYCP3-marked chromosomal axes in WT spermatocytes. (C) Western blot analysis of testicular REC8 protein levels. β-actin loading control shown.


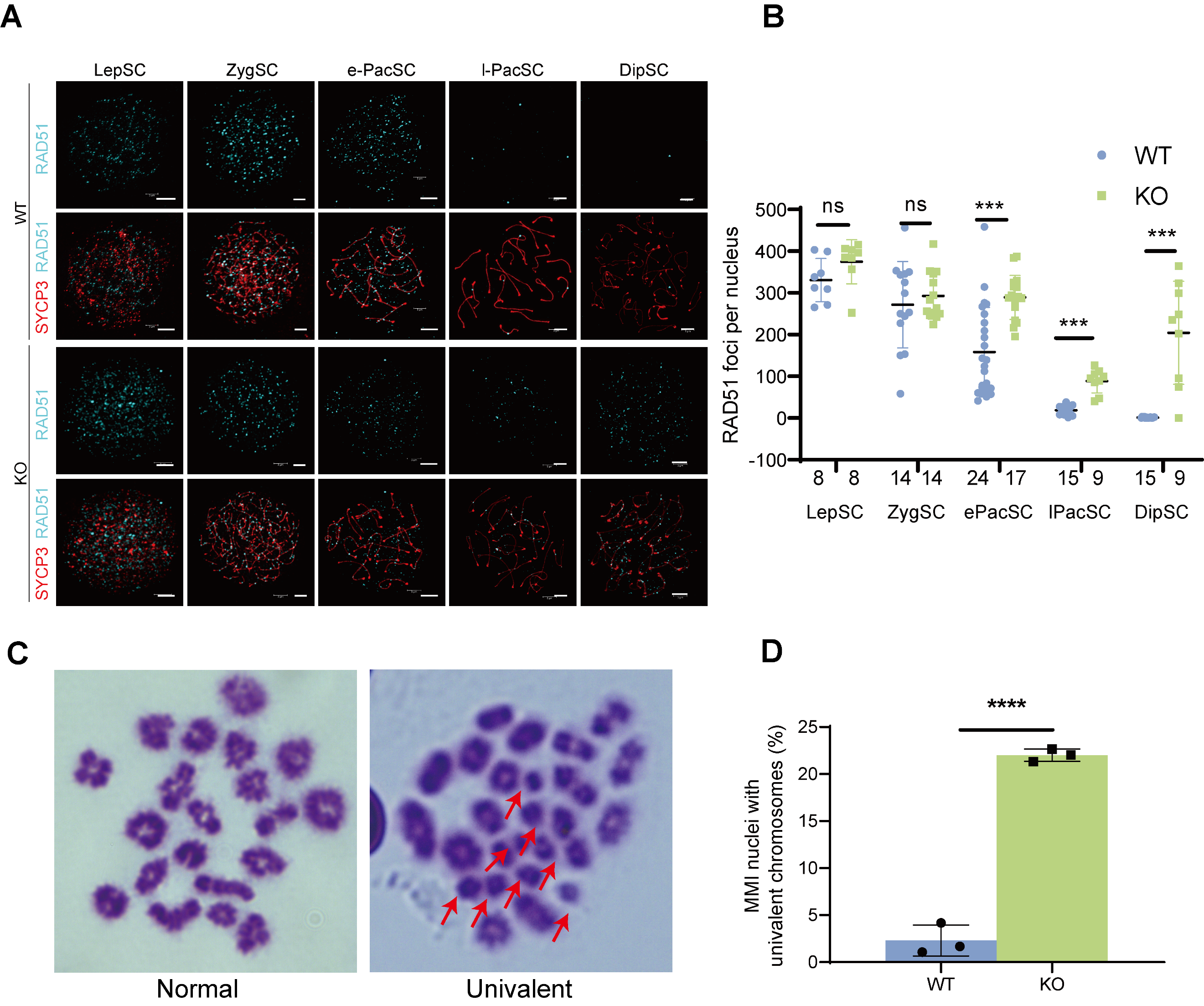


**Figure S4. SOX30 deficiency causes defective HRR and reduced crossover formation.** (A) SYCP3 (red) and RAD51 (recombination intermediates, cyan) co-localization in WT and Sox30 KO spermatocytes. Scale bar: 3 μm. (B) Scatterplot quantifying RAD51 foci counts across meiotic stages. X-axis numbers indicate cells from 3 mice for per stage. (ns: not significant, ***p < 0.001, non-parametric Mann-Whitney U test). (C) Representative metaphase I spreads from control (left) and KO (right) mice, with arrows indicating univalents in the KO cell. (D) The percentage of metaphase I cells with univalents is significantly increased in KO testes (n=3, ****p < 0.0001, non-parametric Mann-Whitney U test).


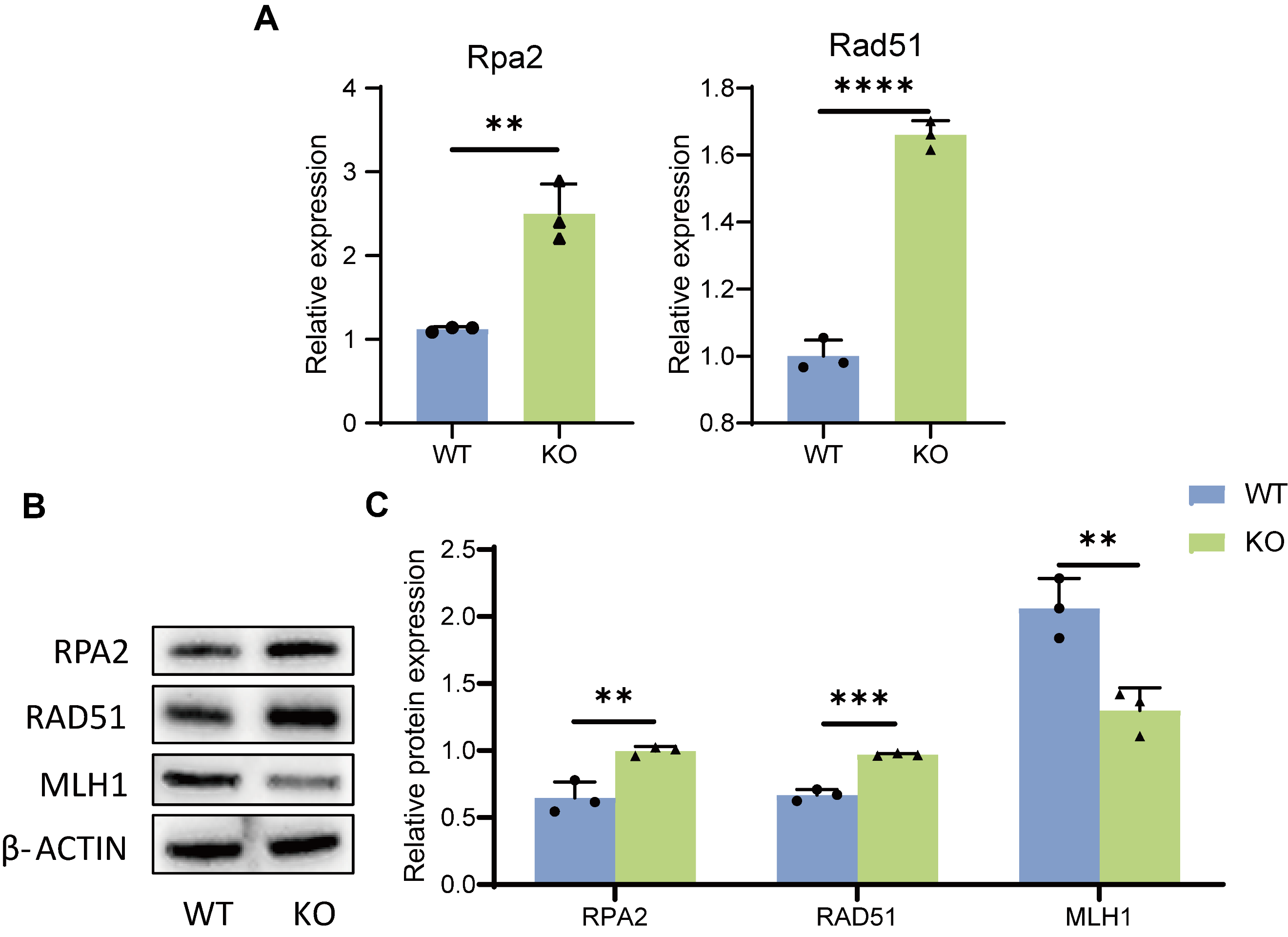


**Figure S5. SOX30 deficiency alters the expression of key HRR factors.** (A) qPCR analysis shows mRNA expression changes of Rpa2 and Rad51 (n=3). (B) Representative western blots of indicated HRR proteins. (C) Quantitative analysis of protein levels from (B) (n=3). Data are mean ± SEM. **p < 0.01, ***p < 0.001; ns, not significant (unpaired t-test).


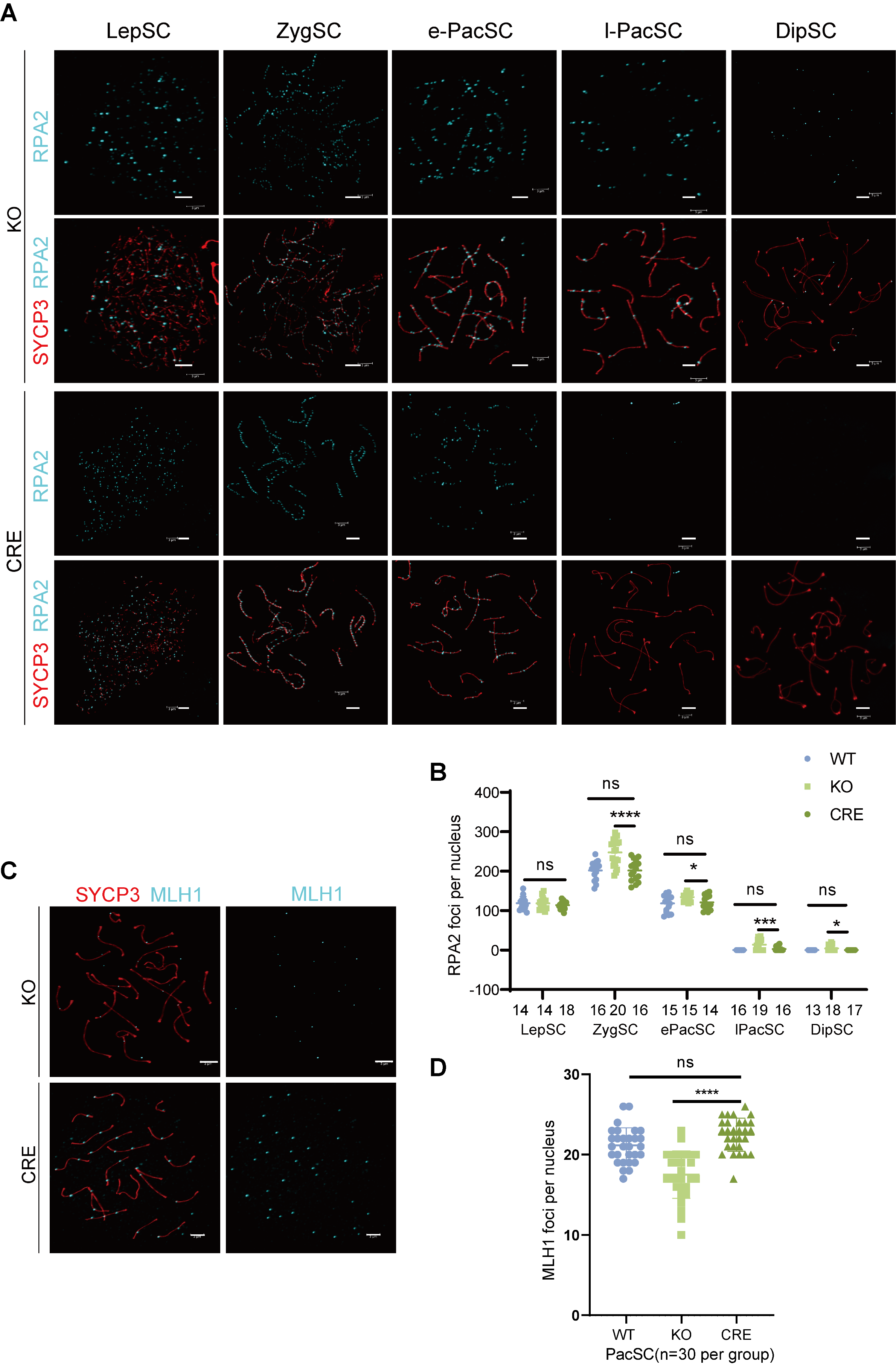
**Figure S6. SOX30 reactivation rescues recombination repair defects in spermatocytes**. (A) SYCP3 (lateral element, red) and RPA2 (recombination intermediate, cyan) co-staining in *Sox30* KO and inducible Sox30-rescued (CRE) spermatocytes. Scale bar: 3 μm. (B) Scatterplot comparing RPA2 foci counts across meiotic stages. Numbers below the x-axis labels indicate cells analyzed per group (ns: not significant, *p < 0.05, *** p < 0.001, ****p < 0.0001, One-way ANOVA test). (C) SYCP3 (red) and MLH1 (crossover sites, cyan) co-localization in *Sox30* KO and CRE spermatocytes. Scale bar: 3 μm. (D) Scatterplot of MLH1 foci counts in pachytene spermatocytes. (ns: not significant, ****p < 0.0001, One-way ANOVA test).


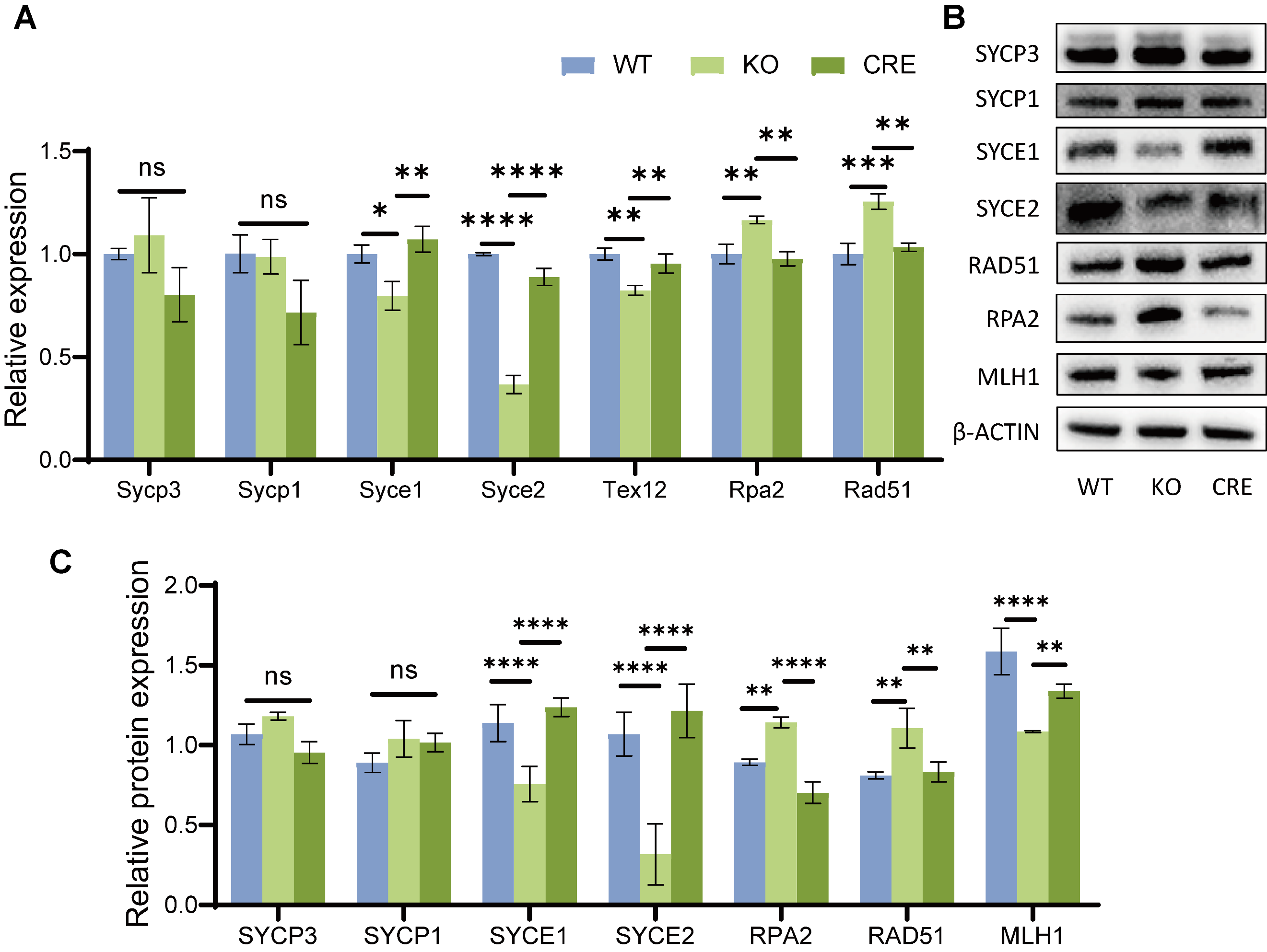


**Figure S7. SOX30 reactivation rescues the expression of key meiotic genes.** (A) qPCR analysis of synaptonemal complex and HRR gene mRNAs (n=3). (B) Representative western blots of SC and HRR proteins. (C) Quantitative analysis of protein levels from (B) (n=3). *p < 0.05, **p < 0.01, ***p < 0.001; ns, not significant.


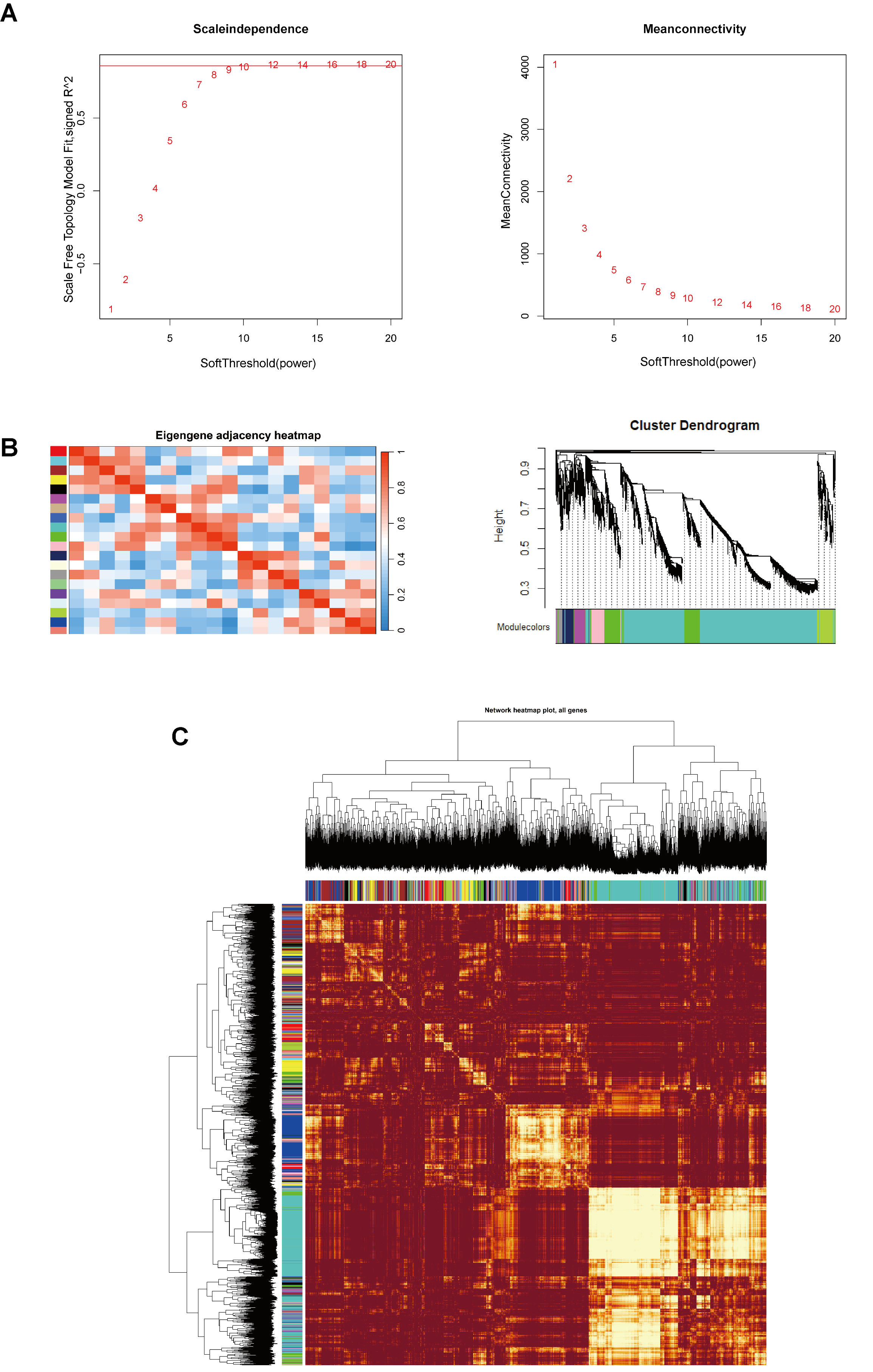


**Figure S8. WGCNA identifies SOX30 as a hub gene in co-expression networks associated with non-obstructive azoospermia (NOA).** (A) Soft threshold power selection for weighted gene co-expression network construction. A power value of β = 12 was chosen based on scale-free topology fit (R² > 0.9) and mean connectivity criteria. (B) Left: Eigengene adjacency heatmap showing pairwise correlations between co-expression modules. Right: Cluster dendrogram of genes with dissimilarity cut defining 18 distinct modules. (C) Network heatmap plot depicting topological overlap matrix (TOM) for all genes. Genes within the turquoise module form a densely interconnected subnetwork.


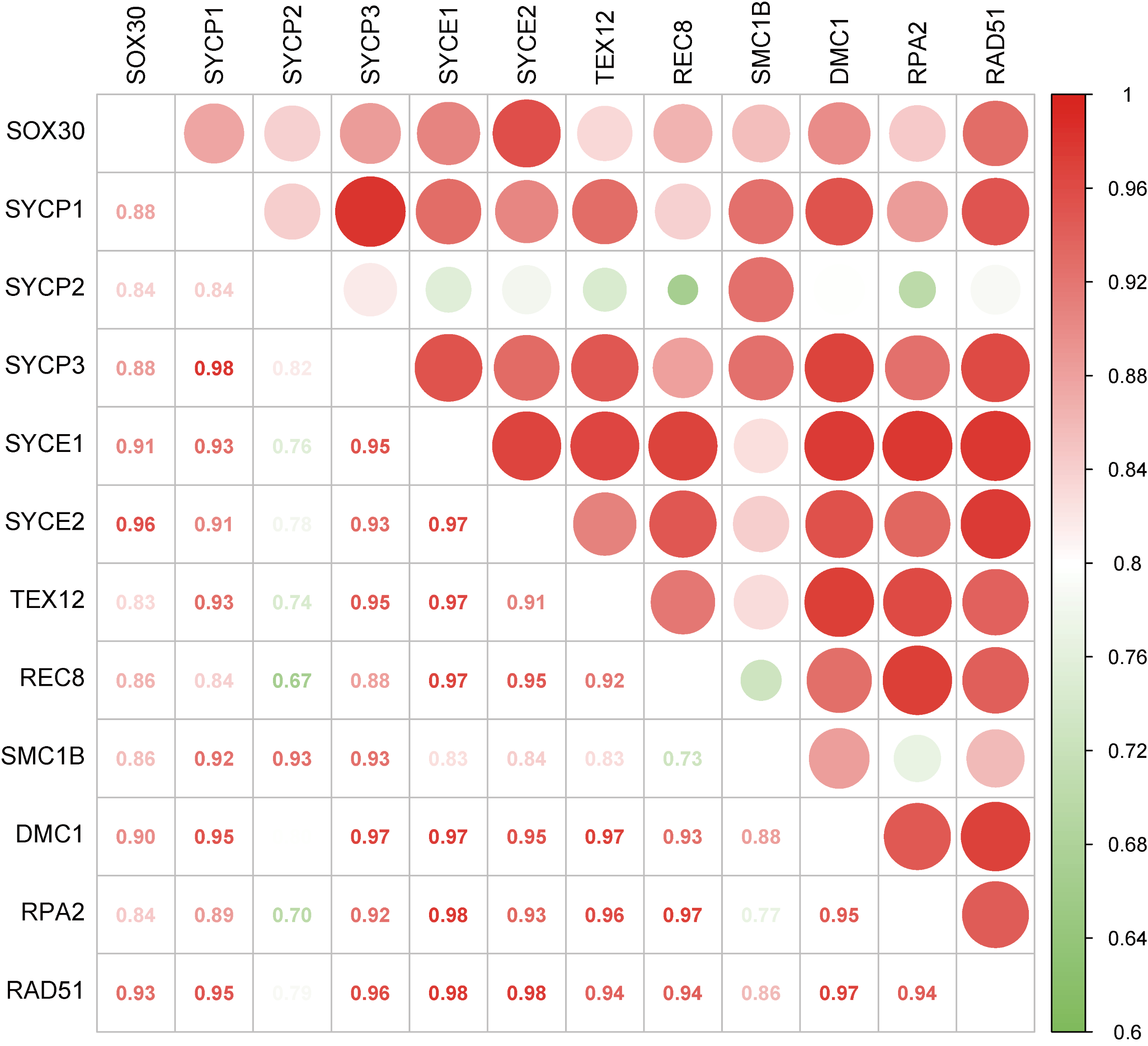


**Figure S9. Correlation Heatmap of SOX30 and Synapsis/Homologous Recombination Repair-Related Genes in Testicular Transcriptome Data from NOA Patients**

**Table S1. Antibodies used in this study**

| **Antibody** | **Rource** | **Identifier** | **Dilution ratio** |
| --- | --- | --- | --- |
| Rabbit polyclonal anti-SOX30 | Abclone | A11759 | WB 1:2000 IF 1:200 |
| Mouse monoclonal anti-SYCP3 | Santa Cruz | sc-74569 | IF 1:20 |
| Rabbit polyclonal anti-SYCP3 | Proteintech | 30079-1-AP | WB 1:1000 |
| Rabbit monoclonal anti-γ-H2AX | Abclone | AP0687 | IF 1:200 |
| Rabbit polyclonal anti-HORMAD1 | Proteintech | 28719-1-AP | IF 1:100 |
| Rabbit polyclonal anti-SYCP1 | Abcam | ab15090 | WB 1:2000 IF 1:200 |
| Rabbit polyclonal anti-SYCE1 | Proteintech | 11063-1-AP | WB 1:2000 IF 1:50 |
| Rabbit polyclonal anti-SYCE2 | Developed at HUABIO |  | WB 1:500 IF 1:20 |
| Rabbit polyclonal anti-TEX12 | Proteintech | 17068-1-AP | IF 1:50 |
| Rabbit polyclonal anti-MLH1 | Proteintech | 11697-1-AP | IF 1:50 |
| Rabbit polyclonal anti-RAD51 | Abcam | ab133534 | IF 1:100 |
| Rabbit polyclonal anti-RPA2 | Abcam | ab76420 | IF 1:50 |
| Rabbit polyclonal anti-REC8 | Abcam | ab192241 | IF 1:100 |
| Goat Anti-Mouse IgG DyLight 488 | Abbkine | A23210 | IF 1:200 |
| Goat Anti-Rabbit IgG DyLight 594 | Abbkine | A23420 | IF 1:200 |
| Goat Anti-Rabbit IgG (H+L) HRP | Zsbio | ZB-2301 | WB 1:5000 |

**Table S2. Primer sequences for qPCR analysis used in this study**

| **Primers** | **Sequences** |
| --- | --- |
| *Sox30* | AACCAAGACATTCTGGCATTGAACT |
|  | CCCATTCCACACTCACACGTCTA |
| *Sycp1* | GAGCAAGAACAGTCATCAGCG |
|  | AGGTGATTCCAGTAAAGATGCCT |
| *Sycp3* | AGCCAGTAACCAGAAAATTGAGC |
|  | CCACTGCTGCAACACATTCATA |
| *Syce1* | GCATGTTGCAGGAGTGTAAAGA |
|  | GCTGCTGTCCAAAACACACATC |
| *Syce2* | TGGACTCTAGCATTGAAACCCT |
|  | TCCTGAATGATTTTGCTGTGGT |
| *Tex12* | TGGCAAACCACCTTGTAAAACC |
|  | TGCTCATATCGCTCAAATCCTTC |
| *Gapdh* | TCCTGGTATGACAACGAAT |
|  | GGTCTCTCTCTTCCTCTTG |

**Table S3. Primer sequences for ChIP-qPCR analysis**

| **Primers** | **Sequences** |
| --- | --- |
| SYCE1 | TAGCATGTTCACGGAAGGTC |
|  | GTGATAGTCGGAGTGAAGACT |
| SYCE2 | GTCTGATGACCTGAGTTGCAT |
|  | TTATAAGCCTCCTGGCATGG |
| SOX30 | TGATTCCAGGGTTCTCGCAC |
|  | CCCCCTTTCGGTTAAGACCC |
